# The Rab5 activator RME-6 is required for amyloid precursor protein endocytosis depending on the YTSI motif

**DOI:** 10.1007/s00018-020-03467-1

**Published:** 2020-02-17

**Authors:** Simone Eggert, Tomas Gruebl, Ritu Rajender, Carsten Rupp, Bianca Sander, Amelie Heesch, Marius Zimmermann, Sebastian Hoepfner, Hanswalter Zentgraf, Stefan Kins

**Affiliations:** 1 Department of Human Biology and Human Genetics, Technical University of Kaiserslautern, Erwin-Schrödinger-Str. 13, 67663 Kaiserslautern, Germany; 2 MPI of Molecular Cell Biology and Genetics, Dresden, Germany; 3 Present Address: Bird & Bird LLM, Munich, Germany; 4 DKFZ, Heidelberg, Germany

**Keywords:** Basolateral sorting signal, Trafficking, Sorting, Clathrin-dependent endocytosis, GDP–GTP exchange factor, Protein interacting with APP tail 1

## Abstract

Endocytosis of the amyloid precursor protein (APP) is critical for generation of β-amyloid, aggregating in Alzheimer's disease. APP endocytosis depending on the intracellular NPTY motif is well investigated, whereas involvement of the YTSI (also termed BaSS) motif remains controversial. Here, we show that APP lacking the YTSI motif (ΔYTSI) displays reduced localization to early endosomes and decreased internalization rates, similar to APP ΔNPTY. Additionally, we show that the YTSI-binding protein, PAT1a interacts with the Rab5 activator RME-6, as shown by several independent assays. Interestingly, knockdown of RME-6 decreased APP endocytosis, whereas overexpression increased the same. Similarly, APP ΔNPTY endocytosis was affected by PAT1a and RME-6 overexpression, whereas APP ΔYTSI internalization remained unchanged. Moreover, we could show that RME-6 mediated increase of APP endocytosis can be diminished upon knocking down PAT1a. Together, our data identify RME-6 as a novel player in APP endocytosis, involving the YTSI-binding protein PAT1a.

**Electronic supplementary material:**

The online version of this article (10.1007/s00018-020-03467-1) contains supplementary material, which is available to authorized users.

## Introduction

The amyloid precursor protein (APP) plays a central role in Alzheimer’s disease (AD), as it is processed into different proteolytic products, including the toxic Aβ peptide, accumulating in AD brain tissue. For Aβ generation, APP is first cleaved by an extracellular sheddase. The resulting membrane stub is subsequently proteolytically converted by the γ-secretase complex. The first step of APP cleavage is mediated by different secretases, including meprin and α-, β- and η-secretases [1]. They all differ by their specific cleavage sites in the APP extracellular domain and mostly process APP at the cell surface [see current review in 2]. However, the major pathogenic step in APP processing is mediated by β-secretase, BACE1, which preferentially takes place in endosomes [3, 4]. Therefore, regulation of APP endocytosis is central for understanding APP pathogenicity.

Similar to many other type I transmembrane proteins, APP passes through the secretory pathway to the plasma membrane [1]. On its way and after it has reached the cell surface, APP is either shed by different secretases or rapidly endocytosed (within minutes), predominantly via clathrin-mediated endocytosis (CME) [5, 6]. Notably, besides CME, APP can also be endocytosed via clathrin-independent pathways, such as the caveolin-mediated internalization [7]. From sorting endosomes, APP is transported to diverse intracellular targets (Golgi apparatus, recycling vesicles, late endosomes, lysosomes and multivesicular bodies). The different endocytic routes are controlled by sorting receptors including the ESCRT (Endosomal Sorting Complex Required for Transport) and retromer complex, as well as different sorting molecules, such as SorLA, SorCS1, calsyntenin and LRP1, and different adaptor proteins interacting with the APP C-terminus [1].

APP displays two sorting signals within its intracellular domain: the YTSI motif, also named basolateral sorting signal (BaSS) [8, 9], and the YENPTY motif [6]. Exchange of the transferrin receptor endocytosis signals by the C-terminus of APP restored transferrin receptor endocytosis and tyrosine to alanine mutations in the NPTY or YTSI sequence were crucial for its endocytosis [8]. Furthermore, deletion of the APP C-terminus in cellular assays [10] or deletion of the last 15 amino acids in mice [11] caused a clear reduction in APP endocytosis. The YENPTY motif is followed by the YKFFE motif, shown to mediate binding of the adaptor proteins AP2 and AP4, allowing APP transport from clathrin-coated vesicles derived from the trans-Golgi network (TGN) to endosomes via the μ4 subunit of the AP4 complex [12]. This motif is also involved in transport from clathrin-coated vesicles derived from the plasma membrane to endosomes, and further to autophagosomes via the AP2/PICALM complex [13]. Moreover, the YENPTY motif interacts with phosphotyrosine-binding (PTB) domain-containing adaptor proteins, such as disabled homolog 2 (Dab2) or protein numb homolog (Numb), mediating APP internalization and formation of clathrin-coated vesicles [14–17].

The role of the YTSI motif in endocytosis remains less clear. It was previously reported to be involved in APP endocytosis [8] as well as in basolateral sorting in polarized Madin–Darby canine kidney (MDCK) cells [9]. Based on these studies, the YTSI motif was designated as a basolateral sorting signal (BaSS). In MDCK cells, basolateral sorting of APP is mediated by the AP-1B complex, which binds the tyrosine residue of the BaSS via its μ1B subunit [18]. However, a mutation of the YTSI motif to ATSI neither affected the APP endocytosis rate, measured via uptake of the radioiodinated APP antibody 1G7 [19], nor sAPPα production [20]. Further, it was reported that basolateral sorting signals have different functions depending on their localization within the protein: motifs located 10–40 amino acids from the transmembrane domain (TMD) are purely endocytic, whereas those located 6–9 amino acids from the TMD normally initiate lysosomal targeting [21]. The YTSI motif of APP is only four amino acids apart from the TMD and was, therefore, assumed to be non-functional in endocytosis [8].

APP can be mono- or multi-ubiquitinated in the C-terminus via at least three of five different lysine residues (K649, K650, K651, K676, K688) [22, 23], affecting its sorting including endocytosis [22, 24–28], possibly as analyzed for epidermal growth factor receptor (EGFR) [29]. How the different endocytosis signals in APP are linked together and which motifs contribute to a higher or lower extent are unknown.

Here, we used detailed imaging analyses and an antibody uptake assay to directly compare the impact of the different endocytosis signals within the APP C-terminus on its internalization. Our data showed that the endocytosis of APP was significantly inhibited by the deletion of the YTSI motif, identical to that observed upon deletion of the NPTY motif, whereas inhibition of ubiquitination only showed a minor influence. Furthermore, we provide clear evidence that the YTSI binding scaffolding protein, protein interacting with APP tail 1a (PAT1a) [30, 31], interacts with the Rab5 activator RME-6, involved in clathrin-dependent endocytosis [32].

## Materials and methods

### Cell lines

N2a cells were maintained in culture media (MEM supplemented with 10% fetal bovine serum, 1% l-glutamine, 1% non-essential amino acids, 1% sodium pyruvate and 1% penicillin/streptomycin). N2a cells were not further differentiated with retinoic acid. HeLa Kyoto cells were maintained in culture media (Dulbecco’s modified Eagle’s medium supplemented with 10% fetal bovine serum, 1% l-glutamine, and 1% penicillin/ streptomycin).

### Plasmids

Expression vectors for the N-terminal myc-tagged human APP mutants lacking the YTSI motif (APP ΔYTSI; deletion of amino acids 652–656 (with reference to APP_695_), the NPTY motif (APP ΔNPTY; deletion of amino acids 680–691), the YTSI as well as the NPTY motif (APP ΔY/N), or the entire APP C-terminus (APP ΔCT; deletion after amino acid 648) were generated by PCR-based mutagenesis, using myc APP in pCEP4 [33] as a template. Generation of N-terminally HA-tagged APP695 WT has been described [33]. Site-directed mutagenesis was used to generate expression vectors for N-terminally HA- and myc-tagged APP 5R, carrying five lysine to arginine exchanges: K649R, K650R, K651R, K676R, K688R. All constructs were validated by double-stranded sequencing. pcDNA3.1+ encoding HA- and His-tagged ubiquitin was kindly provided by J. Riemer (University of Köln, Germany).

For the yeast two-hybrid system, the pHybLex gephyrin and pPC86 DLC1 constructs, as well as the adult mouse brain cDNA library cloned into pPC86, were kindly provided by J. Kirsch (Heidelberg) [34]. Further, cDNAs encoding human PAT1a (1-582), PAT1a-NT (1-278), PAT1a-CT (272-582), and PAT1a-MD (272-359) were cloned in frame in the pHybLex plasmid and the C-terminus of human APLP2 in pPC86. The following PAT1a binding partners were identified: RME-6 (receptor mediated endocytosis-6) = Gapvd1 (GTPase activating protein and VPS9 domains 1) [*Mus musculus* (house mouse)], gene ID 66,691, Pr65/PP2A (protein phosphatase 2, regulatory subunit A, alpha) [*Mus musculus* (house mouse)], gene ID 51,792, snapin/snapap (SNAP-associated protein) [*Mus musculus* (house mouse)], gene ID 20,615, Krba1 (KRAB-A domain containing 1) [*Mus musculus* (house mouse)], gene ID 77,827, Sf3b155 (splicing factor 3b, subunit 1) [*Mus musculus* (house mouse)], gene ID 81,898.

For GST pulldown analyses, GST fusion proteins were generated by cloning the cDNAs of the putative PAT1a interaction partners, identified in the two-hybrid system from the respective pHybLex plasmids in frame in pGEX4T1 (pGEX 2TKN sf3b155 1-598, pGEX 2TKN RME-6 561-1170, pGEX 2TKN Krba1 514-1044 and pGEX 2TKN PP2a/PR65 268-590). pGEX4T1 including the cDNAs encoding the C-terminus of APP, APLP1 or APLP2 have been described previously [31]. N-terminally Flag-tagged hRME-6 in vector pcDNA3.1+ generated via PCR, as well as C-terminally HA-tagged PAT1a in vector pcDNA3.1+ [31], was used for co-transfections in antibody uptake assays.

### Antibodies

The monoclonal rat anti-HA and rat anti-c-myc antibodies were purchased from Hoffmann-La Roche and the mouse anti-c-myc (9E10) antibody was purchased from Santa Cruz Biotechnology. Alexa 488 conjugated mouse monoclonal myc antibody (clone 9E10) was from Millipore. The polyclonal rabbit anti-c-myc antibody was obtained from Synaptic Systems. The mouse β-tubulin antibody and the mouse β-actin antibody were from Sigma. The monoclonal antibody against APP (22C11) has been described before [35]. Rabbit polyclonal antibodies against APLP1 (CT-11) and APLP2 (DII-2) were obtained from Calbiochem. Rabbit and mouse anti-Flag antibodies were purchased from Invitrogen and Sigma-Aldrich, respectively. Rabbit and mouse (early endosome antigen 1 (EEA1) antibodies were obtained from New England Biolabs and BD Biosciences, respectively. Mouse anti-calnexin, anti-n-cadherin, and anti-syntaxin-6 were from BD Biosciences. Rabbit anti-Integrin β-1 was obtained from Epitomics.

For generation of an anti-PAT1a antibody, rabbits were immunized with a synthesized peptide, corresponding to the C-terminal residues 542–572 of human PAT1a. The resulting antiserum was affinity purified using the same peptide (SulfoLink kit, Pierce). For immunoblotting, secondary anti-rabbit, anti-mouse IgG antibodies (Promega), and anti-rat IgG antibody (DAKO Diagnostic), conjugated to horseradish peroxidase were used. For immunofluorescence analyses, secondary anti-mouse, anti-rat and anti-rabbit antibodies were purchased from Molecular Probes (Alexa Fluor series). For generation of monoclonal antibodies against RME-6, the RasGAP-like domain (79-454) of human RME-6 was fused to GST (pGex4T1 hRME6_79-454_) and expressed in BL21 (pLys, DE3). After affinity purification on Glutathione Sepharose beads, the GST-tag was removed and injected in vitro into mice, as described before [36]. Cell culture supernatants from isolated hybridoma cell clones were tested by ELISA, using the recombinant hRME6_79-454_. Following Protein-G Sepharose affinity purification of antibodies from the different positive clones (62s, 67s, 79w and 81s), the antibody solutions were dialyzed against PBS, and the immunoglobulin concentration was adjusted to ~ 4 mg/ml each.

### Antibody uptake assay

The antibody uptake assay was performed as described before [37]. Briefly, N2a cells were cultured in standard MEM media and transfected 24 h after plating (70,000 cells per 14 mm coverslip (Marienfeld)) with jetPRIME (PolyPlus), according to the manufacturer’s instructions, with cDNAs encoding for different N-terminally myc-tagged APP expression vectors. Approximately 20 h post-transfection, cells were placed on ice (to stop endocytosis) and incubated for 30 min with the primary antibody mouse c-myc in OptiMEM (Gibco). After removing unbound antibodies, endocytosis of bound antibodies was allowed at 37 °C, for different time periods in pre-warmed N2a growth media. To stop endocytosis, cells were cooled down to 4 °C and fixed in 4% PFA (Sigma). To visualize residual plasma membrane bound mouse c-myc antibodies, non-permeabilized cells were stained with Alexa Fluor 488-conjugated anti-mouse secondary antibodies. After permeabilization for 10 min with 0.1% NP-40 in 1 × PBS, the cells were blocked with 5% goat serum in 1 × PBS and incubated with Alexa Fluor 594-conjugated anti-mouse secondary antibody to stain remaining surface bound and internalized primary antibodies. Alternatively, the assay was performed using only one secondary antibody (Alexa Fluor 488), to visualize both cell surface and internalized myc APP. One further condition was the performance of the endocytosis assay after co-transfection of APP and its mutants with either Flag RME-6 or PAT1a HA. In this regard, a further incubation step with primary antibody Flag (rabbit) or HA (rat) followed after the permeabilization and blocking step for 1 h at room temperature (RT). For this purpose, additional secondary antibodies, mouse Alexa Fluor 594 (to detect internalized APP), and either rabbit Alexa Fluor 647 or rat Alexa Fluor 647 (to visualize Flag RME-6 or PAT1a HA) were added, respectively. The fourth alternative of this assay involving overexpression of RME-6 included knockdown of PAT1a with an siRNA (Dharmacon) (ID numbers: J-059477-10 and J-059477-12) [38], 48 h prior to the start of the experiment.

The images were taken with the Axio Observer Z.1 Microscope (with Apotome 1; Zeiss). Projections of the z-stacks were performed using ImageJ. Alexa Fluor 488 staining was used to set the ROI for the inner and outer areas of the cell. For quantification, internal/total intensity ratios (*R*_Endo_) of Alexa Fluor 594 immunoreactivity (in case only one secondary antibody was used: Alexa Fluor 488) were determined after endocytosis for different time points (5–20 min). *R*_Endo_ was taken as a quantitative measure of endocytosis rate.

To block endocytosis, cells were treated with the dynamin inhibitor (Dynasore, Sigma-Aldrich), for 30 min in DMEM media without FBS, at 37 °C and 5% CO_2_, at a concentration of 80 µM, prior to starting the antibody uptake assay. In parallel, control cells were treated with the vehicle control, DMSO. 80 μM Dynasore and DMSO were also added during the first antibody incubation at 4 °C, as well as during the internalization time points at 0, 5, 10, and 20 min, in prewarmed N2a media.

### Live cell imaging

For live cell imaging analysis, N2a cells were seeded at a density of 2 × 10^5^ cells in 2 ml N2a media on PLL (Sigma)-coated FluoroDishes (WPI, World Precision Instruments). The cells were transfected the following day with jetPRIME (Polyplus) according to the manufacturers` instructions. After 15–20 h, for analysis of N-terminally myc-tagged APP WT or its endocytosis-deficient mutants, one FlouroDish with transfected cells was placed on ice to stop endocytosis. The cells were washed twice with ice cold Opti-Mem media (Gibco). Subsequently, Opti-Mem was added including Alexa 488 conjugated mouse monoclonal myc antibody (1: 500) (Millipore, clone 9E10) for 30 min at 4 °C to label myc-tagged APP with fluorophore 488 at the cell surface. Afterward, the cells were washed 3 × with ice cold Opti-Mem media to remove unbound myc-488 antibody. Then, prewarmed (37 °C) Opti-Mem media were added to the cells to allow endocytosis. The cells were imaged immediately in the 37 °C/5% CO_2_ Live cell chamber of the microscope using the software Zen 2 pro and the HxP as a light source [microscope Axio Observer Z.1 (Zeiss, Jena, Germany)]. The recordings were performed without a z-stack over a time span of 20 min with intervals of 1 min and exposure times of 500–1000 ms using the definite autofocus function.

Alternatively, myc APP WT-transfected N2a cells were treated pharmacologically for 30 min at 37 °C in plain DMEM media with the inhibitor of endocytosis Dynasore (80 µM) (Sigma-Aldrich) and or the ADAM10 inhibitor GI254023X (10 µM) (Sigma-Aldrich) before starting the endocytosis assay. DMSO (Applichem)-treated cells were used as a control. The pharmacological treatment was continued during the myc-488 antibody labeling step at 4 °C and the 20 min live cell recording at 37 °C (see above). Blinded live cell recordings of 1, 10 and 20 min were analyzed with ImageJ (Wayne Rasband, National Institute of Health, USA).

### Immunocytochemistry

HeLa cells were seeded at a density of 35,000 cells per 24-well plate (Greiner) on 14-mm coverslips and transfected via CaPO_4_. For intracellular staining, the cells were fixed after 18–20 h for 10 min at 37 °C in 4% PFA with 4% sucrose and permeabilized for 10 min with 0.1% NP40. After incubation of primary antibodies at 4 °C overnight and secondary antibodies for 1 h at RT (Alexa Flour 488 and 594 for double staining, Alexa Flour 488, 594 and 647 for triple staining), cells were embedded in Mowiol (Sigma-Aldrich) and subjected to imaging with the software Axiovision 4.8 at the microscope Axio Observer Z.1.

### Quantification of APP localization within early endosomes

For quantification of colocalization of APP within different organelles, specifically designed pipelines for Cell Profiler 2.1.0 [39] were used. Colocalization with the endosomal marker (EEA1 mouse antibody) was based on Pearson correlation, with values ranging from − 1 (perfect exclusion) to 1 (perfect colocalization). A mask of the cell (to define the area of measurement) was drawn in Image J. Output images showing the cell and object segmentations were produced for visual control of the image analysis [40].

### Two-hybrid system

All methods including cDNA library screening and the investigation of protein–protein interactions of known proteins were performed as recommended by Invitrogen in the Hybrid Hunter™, Version F 25-0179 handbook, except that the cDNA library and all prey constructs were provided or cloned in the pPC86 expression vector harboring the GAL4 activation domain (ProQuest™, Invitrogen). For cloning of the bait plasmid, the vector pHybLex/Zeo (Invitrogen) was used. Cloning of bait or prey plasmids was performed using standard PCR-based methods.

### Expression and purification of GST fusion proteins

Expression of GST and GST fusion proteins in *E. coli* and loading of Glutathione Sepharose beads (Amersham Biosciences) were performed as described before [41].

### Coupled in vitro transcription–translation

The in vitro transcription–translation was performed using the in vitro TNT 7 Quick Coupled Transcription-Translation System (Promega), with 1 µg of pcDNA3.1 PAT1a DNA and 250 µCi of [^35^S] methionine for labeling, according to manufacturer’s instructions. Subsequently, in vitro translated PAT1a was incubated with Glutathione Sepharose beads, loaded with target protein fused to GST, in buffer H (50 mM Tris, pH 6.8, 50 mM KCl, 100 mM NaCl, 2 mM CaCl_2_, 2 mM MgCl_2_, 0.1% (w/v) Triton X-100, 5 mM dithiothreitol) for 90 min. After washing three times, the proteins were eluted and analyzed by SDS-PAGE. Then, gels were dried and subjected to autoradiography (MR films, Amersham Biosciences) overnight. Quantification is based on densitometric measurements using ImageJ.

### Co-immunoprecipitation

N2a cells were transiently co-transfected (jetPRIME) with C-terminally HA-tagged PAT1a and Flag-tagged RME-6 WT, RME-6 ΔGAP, and RME-6-ΔVPS9. After 17–24 h, the cells were harvested and lysed in lysis buffer (50 mM Tris/HCl pH 7.5; 150 mM NaCl; 5 mM EDTA; 1% NP40; 1:25 protease inhibitor [Complete (with EDTA), Roche], for 20 min on ice. Equal volumes of cell lysates containing 800 µg protein were precleared with 10 µl Protein A Sepharose (GE Healthcare) for 1 h at 4 °C. After centrifuging the beads for 2 min at 2000×*g* at 4 °C, the supernatant was incubated for 2–3 h with 20 µl HA beads (Roche) at room temperature. Afterward, the beads were washed three times with 750 µl lysis buffer and sedimented for 2 min at 2000×*g*. A last washing step was performed with 10 mM Tris/HCl, pH 7.5. The supernatant was completely removed and the beads were resuspended in 2 × SDS sample buffer (0.125 M Tris/HCl pH 6.8; 20% glycerol; 4% SDS; 0.01% Bromphenol blue; 100 mM DTT). The samples were denatured for 5 min at 95 °C and loaded on an 8% Tris/glycine gel. After Western blotting, Flag-tagged RME-6 and RME-6 deletion constructs were detected with an anti-Flag antibody. Afterward, the same membrane was incubated with antibody HA (3F10) to detect immunoprecipitated PAT1a. Direct loads of the cell lysates were visualized with the same antibodies as the IP samples.

### Transferrin uptake assay

For the transferrin internalization assay, HeLa cells in a 24-well plate were transfected with siRNA specific for RME-6 or control siRNA. At 74 h post-transfection, cells were starved for 4 h in CO_2_-independent DMEM containing 0.2% BSA and then incubated with biotinylated and ruthenium-labeled transferrin at 10 μg/ml, also in CO_2_-independent DMEM containing 0.2% BSA for the indicated time points. Labeling of biotinylated transferrin with the SULFO-TAG-NHS-ester [ruthenium (II) tris-bipyridine, *N*-hydroxysuccinimide; Meso Scale Discovery (MSD)] was performed as suggested by the manufacturer (MSD). After incubation, the cells were placed on ice and the transferrin solution was discarded. Cells were immediately washed with ice cold PBS containing 1 mM CaCl_2_ and 0.5 mM MgCl_2,_ and then washed with DMEM containing 0.2% BSA, pH 2.8, for 2 min (alternatively, 0.5 M NaCl and 0.2 M acetic acid, pH 2.8, were used). Cells were washed again with 1 × PBS and lysed in ice cold lysis buffer (50 mM Tris/HCl, 50 mM NaCl, 1% Triton X-100, 1 × Roche Complete proteinase inhibitor). For lysis, plates were incubated on ice for 5 min and transferred to − 80 °C overnight or for 15 min. After thawing the plates, the lysates were transferred to a 1.5 ml Eppendorf tube, aspirated several times, centrifuged at 13,000×*g* for 10 min at 4 °C, and the cleared supernatant was saved. Three times 40 μl aliquots of each cell lysate containing the internalized biotinylated and ruthenium-labeled transferrin were added to individual wells of a 96-well MSD-High-Bind Multi-Array plate, with carbon electrodes coated with streptavidin, which were previously blocked with 3% MSD Blocker A in MSD Tris wash buffer. The MSD plate was incubated for 1 h at room temperature with agitation. Finally, the cell lysates were discarded, wells were washed once with MSD read buffer T containing surfactant, and the plates were read immediately on a SECTOR Imager 6000 (Meso Scale Discovery). The amount of internalized transferrin was standardized in relation to the total protein concentration of the lysate and expressed as percent of the amount of internalized transferrin in control cells, at the different time points, or comparative relative units.

The mean of three biological repeats, each measured in three individual wells, was calculated and the ± SEM is shown. Since this assay was highly reproducible, error bars are not visible.

### Subcellular fractionation

To perform the subcellular fractionation of post-nuclear cell lysate using an iodixanol gradient, N2a cells expressing myc-tagged APP constructs were lysed in homogenization buffer (300 mM sucrose, 10 mM HEPES pH 7.4, 5 mM EDTA pH 8.0) with protease inhibitor Complete (Roche) with a 27 G needle. Post-nuclear lysates were layered onto a 5–40% continuous iodixanol gradient and centrifuged at 4 °C, 150,000×*g*, for 2 h. 15 fractions of equal volume from the gradient were loaded on an 8% Tris–glycine gel and analyzed via Western blot detection with different primary antibodies. Densitometric measurements of the Western blot signals were performed using Image J.

## Results

### Decreased colocalization of APP endocytosis-deficient mutants and EEA1

**Fig. 1:**
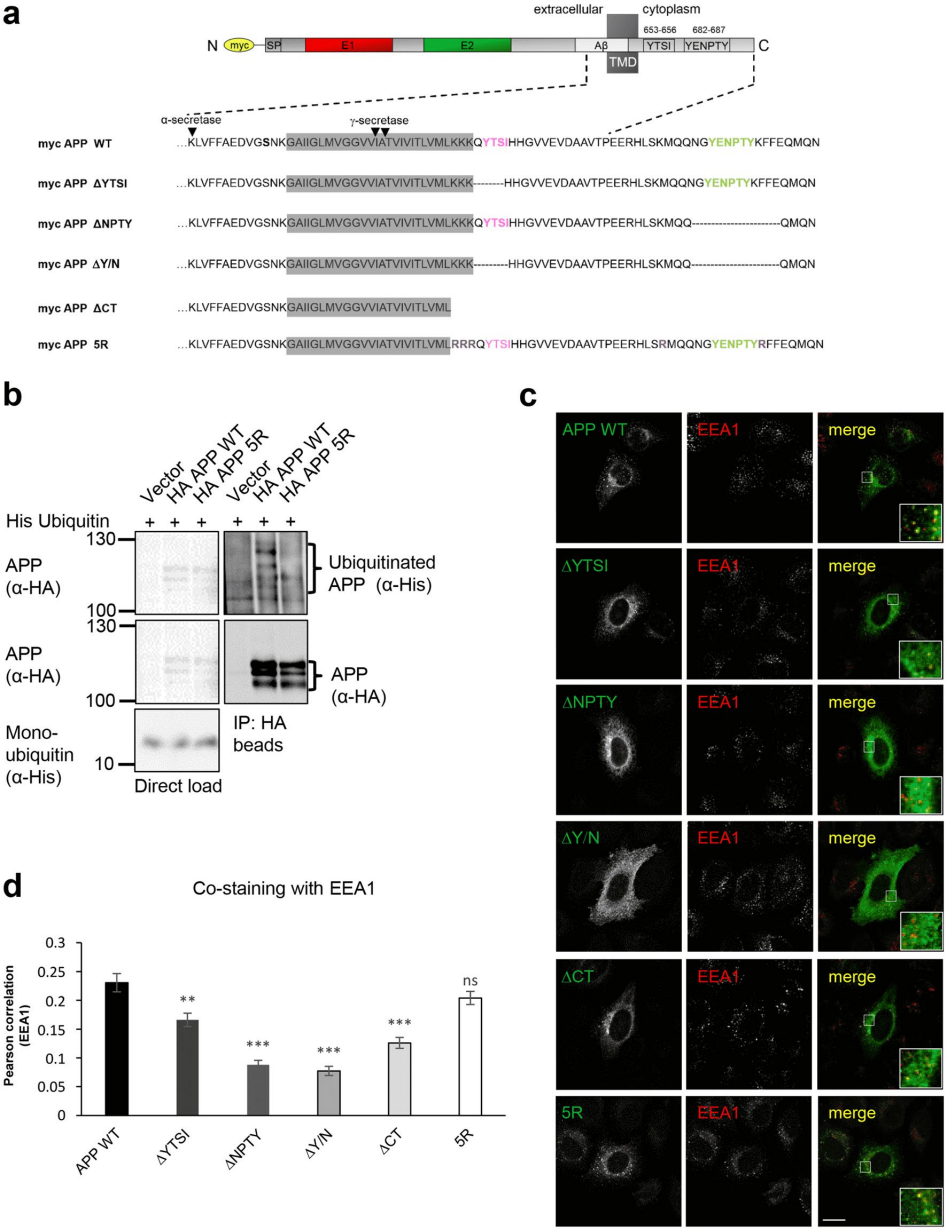
Co-staining of APP deletion mutants and EEA1. **a** Scheme of myc-tagged full-length APP_695_ with the extracellular domain including the E1, E2 and juxtamembranous Aβ domain. For the enlarged C-terminus, the different mutant forms lacking the YTSI (ΔYTSI), NPTY (ΔNPTY), the YTSI and NPTY (ΔY/N), the entire C-terminus (ΔCT) or carrying lysine to arginine substitutions (5R), are shown. **b** Co-immunoprecipitation of APP with ubiquitin. Extracts of N2a cells expressing His-tagged ubiquitin and HA-tagged APP WT, or APP 5R were subjected to immunoprecipitation (IP) with α-HA loaded agarose beads. Western blot analysis of precipitates with α-His and α-HA (3F10) antibodies revealed Co-IP of APP with ubiquitin. Mock transfected cells (Vector) served as a negative control. **c** HeLa cells were transiently transfected via calcium phosphate with N-terminally myc-tagged APP WT, ∆YTSI, ∆NPTY, ∆Y/N, ∆CT or 5R. A cytochemical co-staining using antibodies against APP (c-myc, green) and EEA1 (early endosome antigen 1, red) was performed (scale bar = 20 µm). A 2.5-fold magnification can be observed in the inset (Scale bar = 8 µm). **d** The quantification of the Pearson correlation of APP to EEA1 was calculated via CellProfiler. Bars represent mean values ± SEM, *n* = 10/*N* = 4; unpaired Student’s *t* test: *p < 0.05; **p < 0.01, *n.s.* not significant

To directly compare the influence of the different APP endocytosis motifs on APP internalization, we generated a set of APP mutants lacking either the YTSI motif (APP ΔYTSI) or the NPTY motif (APP ΔNPTY), both the YTSI and NPTY motif (APP ΔY/ N) or the entire APP C-terminus (APP ΔCT) (Fig. 1a). For analysis of the impact of APP ubiquitination on its endocytosis, we generated a ubiquitination-deficient APP mutant, where we substituted all five C-terminal putatively ubiquitinated lysine residues with arginines (K649R, K650R, K651R, K676R, K688R; named APP 5R) (Fig. 1a). All mutants were N-terminally fused to a myc tag for comparable detection. To validate that ubiquitination is indeed impaired in the APP 5R mutant, we co-expressed His-tagged ubiquitin together with HA-tagged APP WT and APP 5R in neuroblastoma (N2a) cells. After precipitation with an anti-HA antibody, we observed a marked reduction of ubiquitinated APP for APP 5R in comparison to APP WT (Fig. 1b), as reported before [24].

To get a first indication if deletion of the different endocytosis motifs in APP affects its subcellular localization, we analyzed their distribution pattern in an iodixanol gradient (0–40%) (Online Resource 1). Interestingly, the pattern for APP WT and the APP 5R mutant appeared similar to each other, whereas APP lacking the YTSI and/or the NPTY motif clearly showed a different distribution pattern, which was even more pronounced in the APP ΔYTSI/ΔNPTY double mutant. This indicates that the APP 5R mutant might affect APP endocytosis less severely. To address this point in more detail, we investigated endosomal localization of the APP mutants via immunocytochemistry with the marker for early endosomes, EEA1. For this purpose, HeLa as well as N2a cells heterologously expressing N-terminally myc-tagged APP WT, ∆YTSI, ∆NPTY, ∆Y/N, ∆CT and 5R were fixed 18 h post-transfection and co-stained using antibodies against APP (c-myc, green) and EEA1 (Early Endosome Antigen 1, red) (Fig. 1c, Online Resource 2). The quantification via Pearson correlation of APP to EEA1 was calculated via CellProfiler. Bars represent mean values ± SEM, *n* = 9/*N* = 4; unpaired Student’s *t* test: *p < 0.05; **p < 0.01, n.s., not significant (Fig. 1d, Online Resource 2). We observed that colocalization with EEA1 was significantly reduced in case of APP mutants lacking either the NPTY or YTSI motif. Notably, APP ∆YTSI showed some vesicular staining, while the vesicular pattern was more severely reduced in APP ∆NPTY, ∆Y/N, and ∆CT. In contrast, the presence of APP 5R in early endosomes was not significantly altered. Together, our data show that the YTSI and NPTY motifs are required for APP endocytic localization.

Notably, we recognized a relatively weak Golgi staining for the APP ∆Y/N mutant (Fig. 1c) and the detected endosomal localization was lower than that observed for APP lacking the entire C-terminus (Fig. 1d). This could indicate that these mutants perhaps affect additional cellular trafficking steps in the secretory or late endosomal pathway, besides APP endocytosis. However, more detailed studies will be required to clarify this point.

### Additive influence of the YTSI and NPTY motif on APP endocytosis

**Fig. 2:**
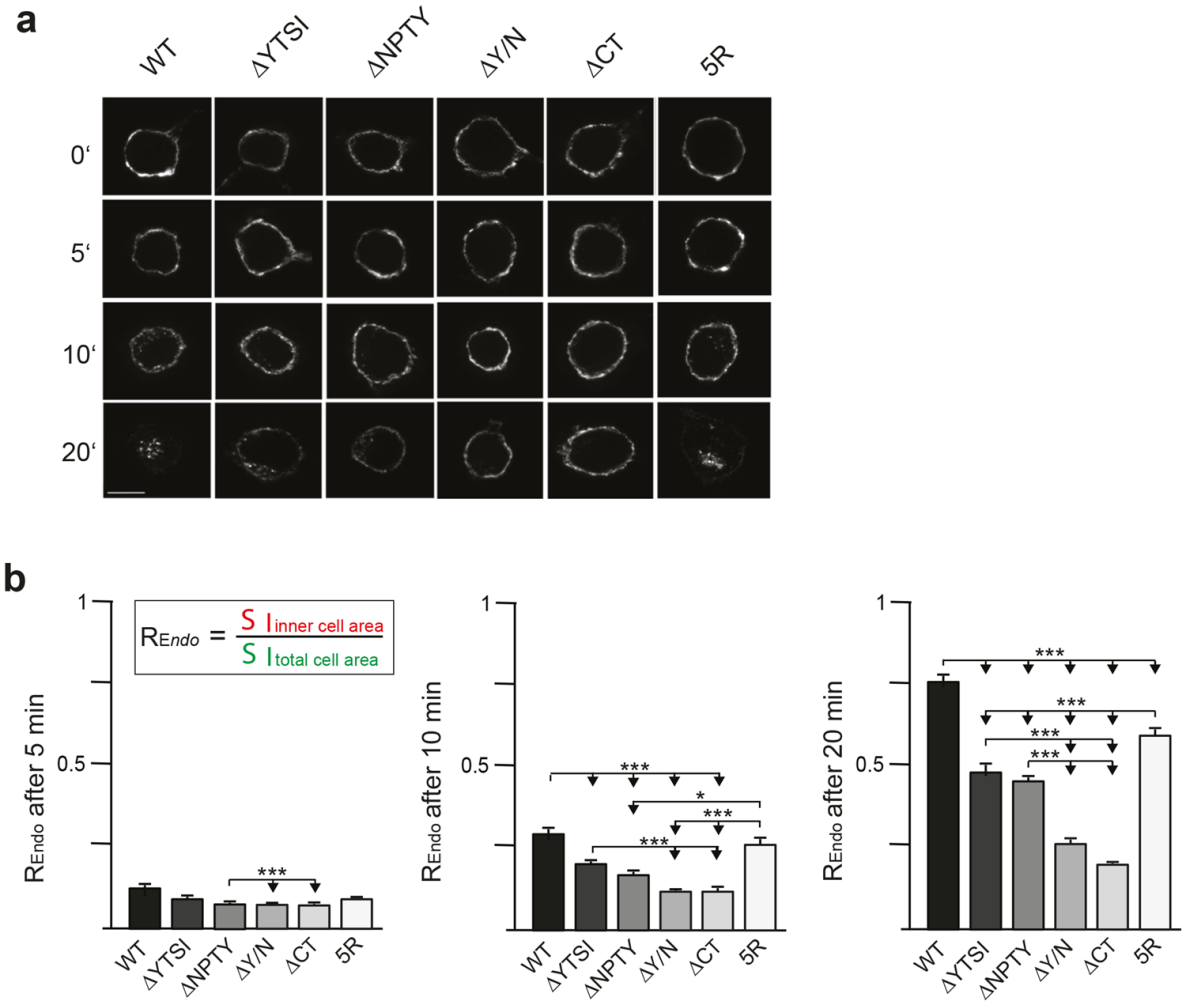
Endocytosis of APP depends mainly on the NPTY and YTSI motif. **a** Antibody-uptake assay. N2a-cells expressing myc APP or the indicated APP mutant variants were incubated with a mouse anti-myc antibody at 4 °C. Subsequently, the cells were incubated at 37 °C, allowing antibody uptake. After 0, 5, 10 or 20 min, cells were fixed and incubated with an anti-mouse Alexa Fluor 488-conjugated secondary antibody to label surface APP. The cells were then permeabilized and stained with an anti-mouse Alexa Fluor 594-conjugated secondary antibody. In this figure, only the signal of Alexa Fluor 594 is shown, representing endocytosed and remaining surface APP. Representative immunofluorescent pictures of N2a cells expressing myc-tagged APP WT, APP ΔYTSI, APP ΔNPTY, APP ΔY/N, APP ΔCT and APP 5R after 0, 5, 10 and 20 min of endocytosis (scale bar: 10 μm). **b** Quantification of the endocytosis rate of APP deletion mutants. Internal/total intensity ratios (*E*_rate_) for APP WT and indicated APP mutants were determined after endocytosis for 5, 10 and 20 min. Bars represent mean values ± SEM; *n* ~ 40 cells; Kruskal–Wallis test with Dunn’s post hoc test. *p < 0.05, **p < 0.01, ***p < 0.001. **c** Quantification of live cell imaging analysis. Antibody uptake assay was performed as described above, but after transfer from 4 to 37 °C, cells were immediately subjected for video microscopic analysis. Quantification of the endocytosis rate of APP WT and indicated APP deletion mutants is shown by internal/total intensity ratios (*E*_rate_) for APP WT and indicated APP mutants. Bars represent mean values ± SEM; *n* ~ 10 cells; ANOVA test with Tukey’s post hoc test. *p < 0.05, **p < 0.01, ***p < 0.001

We assumed that the defect in endocytic localization of APP **∆**YTSI, ∆NPTY, and ∆Y/N was due to reduced endocytosis rates. To validate this assumption, we tested the different N-terminally myc-tagged APP mutants in an antibody uptake assay. All APP variants were individually expressed in N2a cells. 18 h post-transfection, the cells were incubated with an anti-c-myc (9E10) antibody at 4 °C to label surface APP. Then, the cells were moved to 37 °C to allow endocytosis followed by fixation either immediately (0 min) or 5, 10 or 20 min after incubation, at 37 °C. Residual surface APP was detected with a fluorescent secondary antibody Alexa Fluor 488 and. after subsequent permeabilization, endocytosed as well as remaining surface APP were labeled with a different secondary antibody, Alexa Fluor 594. This labeling enabled the differentiation between surface APP and internalized APP at different time points, upon induced endocytosis. Here, only the signal of Alexa Fluor 594 is shown, which represents endocytosed APP and remaining cell surface APP (Fig. 2a). For quantification, the endocytosis rate (Endo) was determined by calculating the ratio of signal intensity of endocytosed APP (immunoreactivity of Alexa Fluor 594 for internal cell) to total intensity of the cell (immunoreactivity of Alexa Fluor 594 for the whole cell including the plasma membrane) (Fig. 2b). After 20 min of endocytosis, APP WT was almost completely internalized. Internalization of APP ΔYTSI and APP ΔNPTY was significantly impaired by about 30–40%, compared to APP WT after endocytosis for 10 or 20 min (Fig. 2b). A lower impairment was observed for the endocytosis rate of APP 5R, which was only reduced by about 20% and reached significance levels only at a time point after 20 min. Interestingly, we observed the strongest inhibition (74%) of endocytosis for APP lacking the entire C-terminus. A similar nearly complete block of endocytosis was achieved by usage of Dynasore (Online Resource 3), indicating that APP endocytosis mostly depends on dynamin-dependent endocytosis mechanisms. Together, these data suggest that the NPTY and YTSI motifs, as well as to a minor extent ubiquitination, contribute to APP endocytosis.

To further validate our findings, we performed live cell imaging of the antibody uptake assay, allowing to follow APP-mediated antibody uptake on individual N2a cells. 15–20 h post-transfection, the cells were incubated with a 488-labeled anti-c-myc antibody at 4 °C to mark surface APP. Then, the cells were incubated at 37 °C and endocytosis was monitored by live cell imaging for 20 min at 1 min intervals (Fig. 2c; videos in Online Resource 4–8). For quantification, the endocytosis rate (Erate) was determined by calculating the ratio of signal intensity of endocytosed intracellular APP to total APP immunoreactivity including the plasma membrane, 1 and 20 min after incubation at 37 °C. All APP mutants were analyzed under the same conditions. After 20 min of endocytosis, significant amounts of APP WT and APP 5R were internalized. In contrast, internalization of APP ΔYTSI, APP ΔNPTY, APP ΔY/N and APPΔCT was clearly impaired (Fig. 2c), in line with the data obtained by immunocytochemical analyses of the antibody uptake assay (Fig. 2a, b).

As APP can be cleaved by α-secretase at the cell surface [42], mainly by ADAM10 [43], this might cause the release of the APP extracellular domain, thus reducing fluorescent signals derived from cell surface APP. Therefore, we tested if contribution of α-cleavage could affect our endocytosis measurements. For this purpose, we performed the antibody uptake assay with APP WT in the presence of an endocytosis inhibitor (Dynasore) together with an ADAM10 α-secretase inhibitor or with DMSO as a control. As observed before, in the presence of Dynasore, cell surface APP immunoreactivity remained unchanged over the entire duration of the assay (20 min) (Online Resource 3). Interestingly, co-incubation with the ADAM10 secretase inhibitor [44] neither significantly affect the APP endocytosis rate, nor cell surface levels in the recorded time period of 20 min compared to Dynasore-treated cells (Online Resource 3, Videos in Online Resource 9, 10). These data show that the antibody uptake assay is relatively insensitive to ADAM10-dependent processing of APP.

All together, these data indicate that the NPTY and YTSI motifs represent the major motifs required for APP endocytosis.

### The YTSI-binding protein PAT1a interacts with the Rab5 activator RME-6

**Fig. 3:**
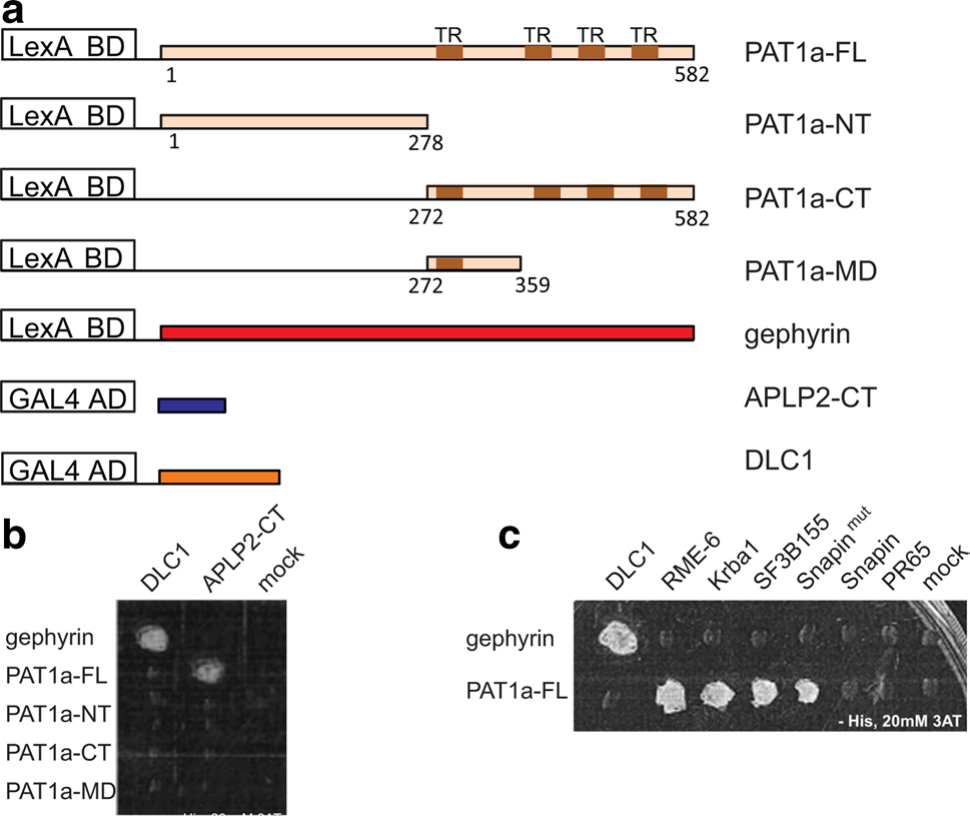
RME-6 is an interaction partner of PAT1a. **a** To set up the yeast two-hybrid system for a screen of novel PAT1a interaction partners, the LexA-DNA binding domain (LexBD) and GAL4 activation domain (GAL4AD) were fused to PAT1a full-length (PAT1a-FL) and different deletion constructs encompassing the N-terminal (PAT1a-NT), the C-terminal (PAT1a-CT) or the middle part of PAT1a (PAT1a-MD) and the C-terminus of APLP2 (APLP2-CT) known to interact with PAT1a [30], respectively. Further, a pair of known interaction partners, gephyrin and DLC1 fused to LexBD and GAL4AD, served as positive control. **b** A yeast two-hybrid screen of PAT1a FL protein and different deletion constructs with APLP2-CT as a positive control, as well as gephyrin with DLC1 as a positive control is shown. **c** From 6.8 × 10^7^ clones of an adult mouse brain cDNA library, the cDNAs of 100 His prototrophic clones were isolated and determined by sequencing. Only five different cDNAs were identified. For validation, the cDNAs were separately re-transfected in L40 yeast cells together with the GAL4AD-PAT1a-FL plasmid and subsequently plated on auxotrophic selection medium lacking histidine and 20 mM 3-aminotriazole (3-AT). Interactions between RME-6 (561-1170), Krba1 (514-1044) (Krueppel associated box A contain protein 1) and sf3b155 (1-598) (splice factor 3b) could be confirmed. Co-expression of PAT1a with the Snapin (1-45 + 28) pulled in the two-hybrid screen also caused reporter gene activation. However, a corrected form of Snapin (Snapin (1-45)), PR65 (268-590), or the AD alone (pPC86) (mock) did not reconstitute transcriptional reporter gene activation, together with PAT1a. Co-transfection of the same cDNAs with GAL4AD–gephyrin as bait served as negative control and co-expression of LexBD–DLC1 and GAL4AD–gephyrin, as positive control

**Fig. 4:**
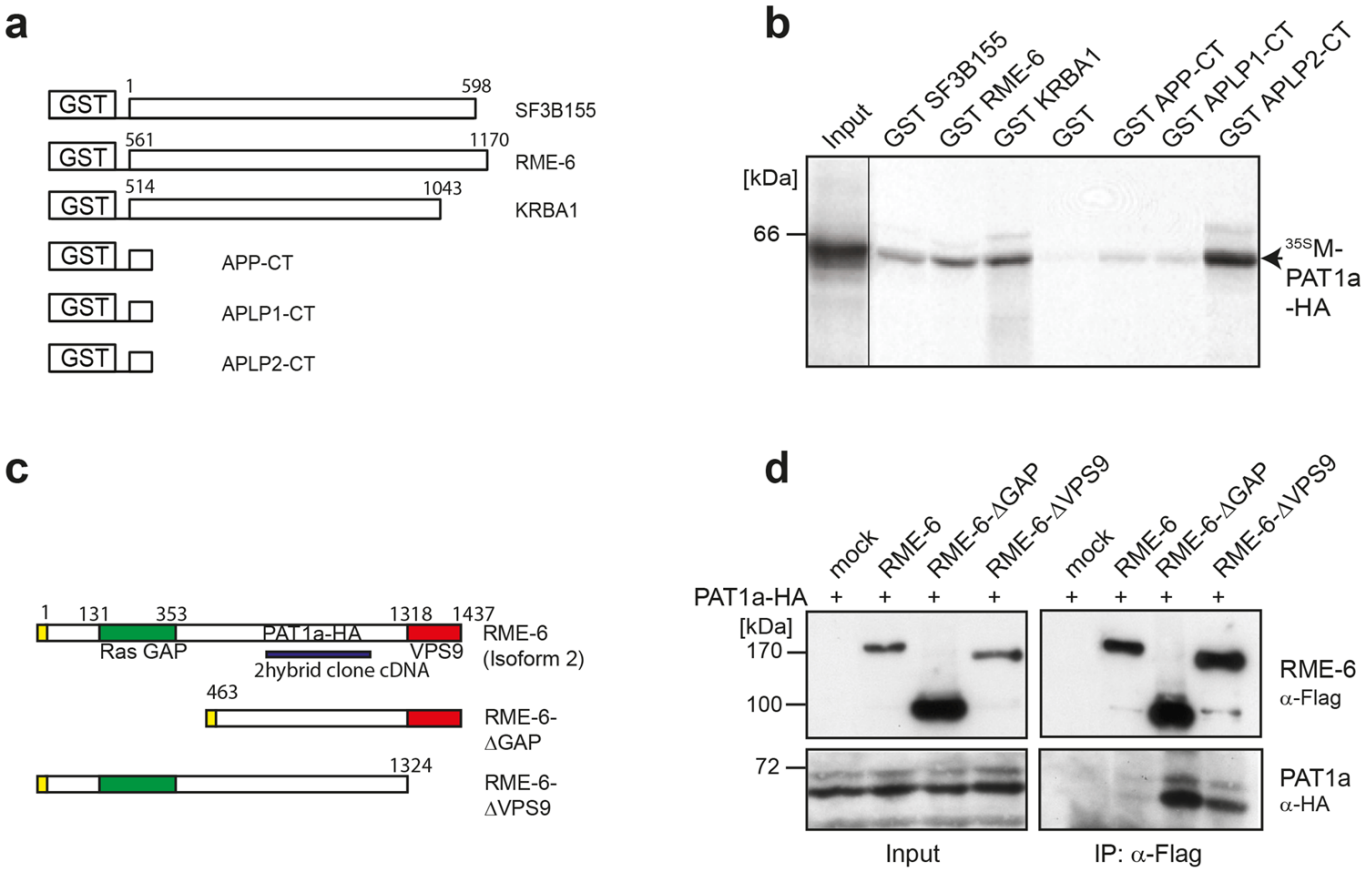
Validation of the PAT1a / RME-6 interaction. **a** Schematic illustration of the corresponding GST fusion proteins. Numbering refers to the corresponding full-length proteins. **b** GST pulldown analysis. Radioactively labeled in vitro translated PAT1a (15% were loaded as Input) was incubated with beads loaded with equal amounts of GST-sf3b155, GST-RME-6, GST-Krba1 or GST alone. Recombinant PAT1a bound specifically to beads loaded with the GST fusion proteins but not to GST alone. As a positive control, GST pulldown analyses were also performed with radioactively labeled PAT1a and GST-APP-CT, GST-APLP1-CT, or GST-APLP2-CT in parallel. Retained, radioactively labeled PAT1a was eluted, separated by SDS-PAGE and monitored by autoradiography. **c** Scheme of Flag-tagged full-length RME-6 (FL) and two mutants, lacking either the N-terminal GTPase activating domain (GAP domain) (ΔGAP) or part of the C-terminal vacuolar sorting protein 9 (ΔVPS9). The PAT1a binding site, as evidenced by yeast two-hybrid and GST pulldown analysis is highlighted by a thick line. **d** Co-immunoprecipitation of RME-6 with PAT1a. N2a cells expressing HA-tagged PAT1a together with Flag-tagged full-length RME6 FL, RME6 ΔGAP or RME6 ΔVPS9 were lysed 24 h post-transfection and subjected to co-immunoprecipitation (IP) with anti-Flag antibodies. Precipitates were analyzed by Western blot with a different anti-Flag antibody and anti-HA antibodies. Cells transfected with empty Flag expression vector served as control (mock). 12% of cell homogenates (input) were loaded for control. Note, much stronger PAT1a binding of RME-6 lacking either the GAP or VPS9 domain. Co-IP experiments were performed three times. Only one representative experiment is shown

As pointed out before, the NPTY motif was shown to mediate clathrin-dependent endocytosis [8, 19], whereas the molecular mechanism underlying the YTSI motif on endocytosis is still unclear. In previous studies, it was demonstrated that PAT1 and the more frequent variant PAT1a interact with the YTSI of APP directly and affect APP trafficking [30, 31]. In an attempt to understand if perturbed binding of PAT1a to APP could explain the altered endocytosis rate of APP ΔYTSI, we performed a yeast two-hybrid screen to identify novel PAT1a interacting molecules, possibly involved in APP endocytosis. As binding of PAT1a is significantly stronger to APLP2 than to APP (data not shown; see Fig. 4b), we decided to work with the APLP2 C-terminus for the screen. To set up the yeast two-hybrid system, we used LexA BD fused to gephyrin and DLC1 fused to the GAL4 AD (Fig. 3a). Consistent with previous studies, co-expression of the two interacting hybrid proteins in yeast L40 strain caused a reconstitution of transcriptional activation of the reporter genes, monitored by growth on auxotrophic agar plates (Fig. 3b) [45]. LexA BD-PAT1a fusion construct harboring aside from full-length PAT1a (PAT1a 1-585) different PAT1a deletion constructs (PAT1a 1-278, PAT1a 272-585 and PAT1a 272-359) (Fig. 3a) showed a low self-activating activity that could be suppressed by 20 mM 3-aminotriazole (3-AT), a competitive inhibitor of imidazole-glycerol phosphate dehydratase. The GAL4 complementation by specific binding of PAT1a-GAL4BD to interaction partners such as APLP2-CT fused to GAL4AD was unaffected in growth on selection medium supplemented with 3-AT (Fig. 3b). Interestingly, only LexA BD fused to full-length PAT1a resulted in histidine prototrophy of the respective yeast transformants, indicating that full-length PAT1a is essential for its interaction with the intracellular domain of APLP2 (Fig. 3b), arguing for a more complex interaction interface between PAT1a and the intracellular domain of APLP2. No growth was observed when the LexA BD-PAT1a was co-expressed with GAL4 AD-DLC1 or when LexA BD-gephyrin was co-expressed with the GAL4 AD-APLP2-CT, validating the specificity of the interaction in the two-hybrid system (Fig. 3b). To identify novel PAT1a-binding proteins, a yeast two-hybrid screen was performed using full-length PAT1a as bait. From 6.8 × 10^7^ clones of an amplified adult mouse brain cDNA library, 100 different His-positive clones were isolated. Sequencing revealed that 86 clones harbor cDNA fragments with frameshifts in the open reading frame. At the end, only 5 out of 14 different cDNAs were identified, encoding: (1) receptor mediated endocytosis-6 (RME-6), first identified in *Caenorhabditis elegans* as a regulator of Rab5-dependent endocytosis [46]; (2) a yet non-characterized transcript, NM_133922 [47], named Krueppel associated box (KRAB-A) domain containing protein 1, Krba1; (3) splice factor 3 subunit b (sf3b) 155 [48, 49]; (4) Snapin/Snapap, a Snap-25 interacting protein [50, 51]. Notably, complete sequencing of Snapin cDNA revealed a small deletion of five nucleotides at position 135, resulting in the shift in the ORF, causing an exchange of the C-terminus of Snapin after amino acid 45–28 artificial amino acids. Therefore, the originally identified clone was named Snapin^mut^. For control, a construct was cloned lacking the artificially generated C-terminal polypeptide (Snapin (1-45)); (5) α-subunit (PR65) of protein phosphatase 2A (PP2A) [52, 53]. All isolated plasmids from the positive clones including Snapin (1-45) were retransformed into yeast together with LexA BD-PAT1a. Reporter gene activation was observed for RME-6, Krba1, sf3b155 and Snapin^mut^ fused to the GAL4 AD domain, validating the interaction with PAT1a in yeast (Fig. 3c). However, only marginal growth of yeast cells co-transformed with PR65/GAL4 or Snapin (1-45) was observed, suggesting that the previously observed activation of the reporter gene was likely unspecific. None of the isolated prey plasmids activated reporter gene transcription alone (data not shown), nor did they interact with the control bait protein gephyrin, demonstrating specificity of the interaction with PAT1a. These data suggest that RME-6, Krba1 and Sf3b are putative novel interaction partners of PAT1a.

From the positive clones, the cDNAs were subcloned from the two-hybrid vector into a glutathione S-transferase expression vector. The GST-RME-6 (561-1170), -Krba1 (514-1044) and -Sf3b (1-598) fusion proteins as well as APP-CT, APLP1-CT, and APLP2-CT [described in 31] fused to GST (Fig. 4a), and for control GST only were expressed in bacteria and affinity purified. GST pulldown analyses were conducted with ^35^S-radioactively labeled, in vitro translated PAT1a. PAT1a was specifically retained on Glutathione Sepharose beads loaded with GST-sf3b, GST-RME-6 and GST-Krba1 (Fig. 4b). No PAT1a was bound to beads loaded with GST alone. Notably, a similar quantity of PAT1a was retained on beads loaded with GST-sf3b155, GST-RME-6 and GST-Krba1, compared to GST-APP-CT or GST-APLP1-CT, arguing for a relatively strong binding of PAT1a to the tested GST fusion proteins. Together, these data validate that RME-6, Krba1 and sf3b interact with PAT1a.

**Fig. 5:**
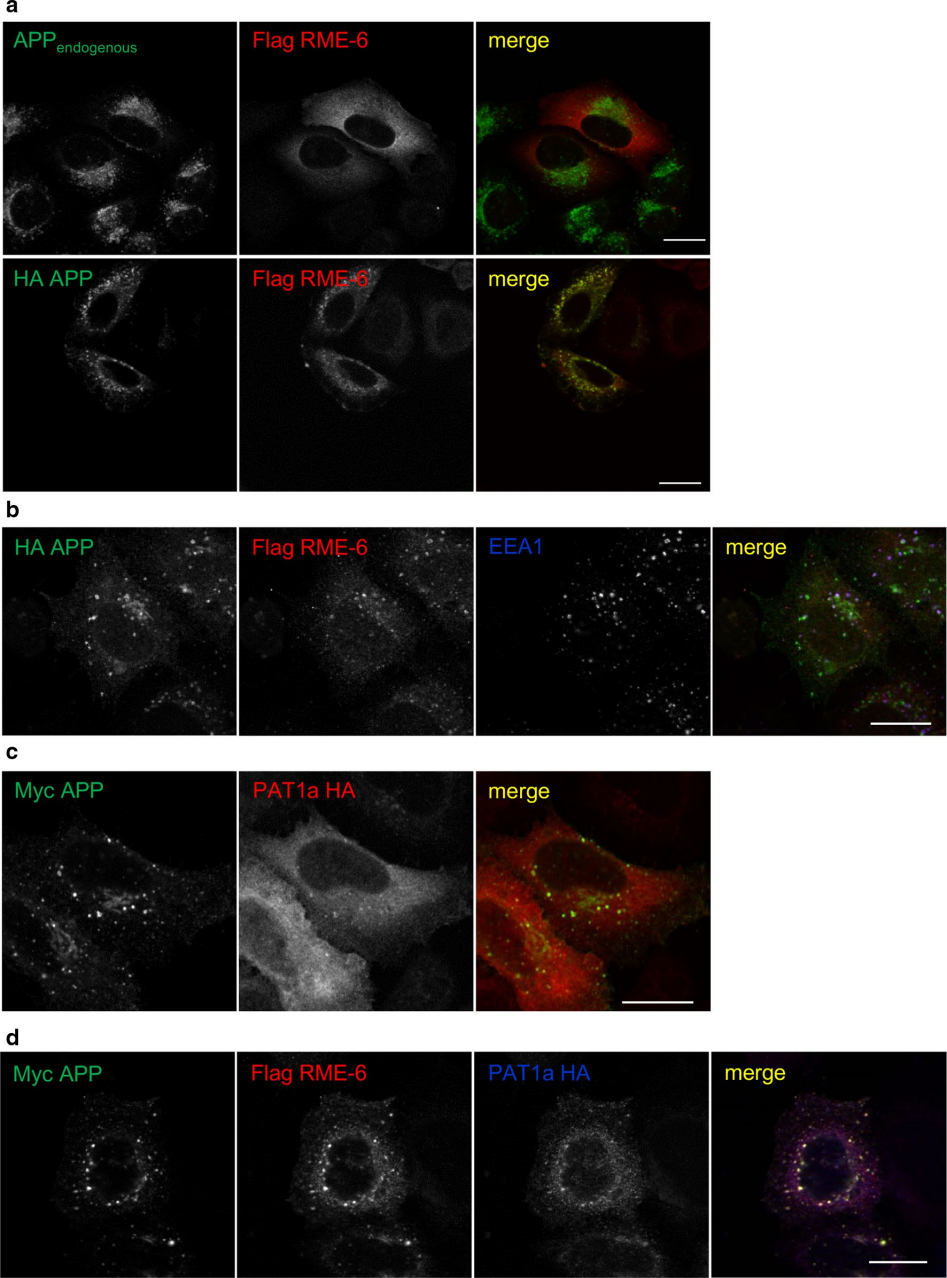
Colocalization of APP, RME-6 and PAT1a. **a** HeLa cells were transiently transfected with Flag RME-6 and immunostained for endogenous APP (Y188, green) and overexpressed RME-6 (Flag-tag, red) (upper panel). HeLa cells were transiently transfected with HA APP_695_ and Flag RME-6. A cytochemical co-staining using antibodies against APP (HA-tag, green) and RME-6 (Flag-tag, red) was performed (lower panel) (Scale bar: 20 µm). **b** HeLa cells co-expressing HA APP_695_ and Flag RME-6 were immunostained with anti-HA (green), anti-Flag (red) and anti-EEA1 (blue) antibody. Overlay of all three channels is indicated in white. **c** Cells heterologously expressing HA-tagged PAT1a (PAT1a HA, red) and myc-tagged APP (myc APP, green) were immunostained (Scale bar: 20 µm). **d** Triple-transfection of HeLa cells with expression constructs encoding myc APP (green), Flag RME-6 (red) and PAT1a HA (blue), were immunostained. Overlay of all three channels is shown in white, suggesting tripartite complex formation. Scale bar: 20 µm

In regard to the observed role of the YTSI motif in endocytosis, we focused our further analyses on the putative interaction of PAT1a with the Rab5 activator RME-6. The corresponding full-length mouse RME-6 cDNA and different RME-6 deletion constructs lacking the N-terminal RasGAP-like domain (RME-6 ΔGAP) or harboring a nonfunctional VPS9 domain (RME-6 ΔVPS9) were cloned in a Flag-tag containing expression vector (Fig. 4c). Flag-tagged full-length RME-6 and RME-6 mutants were co-expressed with HA-tagged PAT1a in mouse neuroblastoma cells. Western blot analysis of the lysates with an anti-Flag antibody revealed that all RME-6 forms were expressed efficiently and displayed the expected apparent molecular weight. Co-immunoprecipitation experiments with an anti-Flag antibody (M2) revealed weak binding of PAT1a to RME-6. The binding affinity between HA-PAT1a and Flag RME-6 could be slightly increased by treating the cell lysate with shrimp alkaline phosphatase prior to co-immunoprecipitation (data not shown), suggesting a minor impact of Ser/Thr- phosphorylation on PAT1a/RME-6 complex formation. Notably, a strong increase in the amount of co-immunoprecipitated PAT1a was observed for both RME-6 deletion constructs, RME-6 ΔGAP and RME-6 ΔVPS9, when compared to full-length RME-6. This can be explained by a structural change in RME-6 after deletion of either the GAP or VPS9 domain. As a negative control, Co-IP experiments were performed with N2a cells co-transfected with empty vector (mock) and HA-tagged PAT1a (Fig. 4d**)**. Together, the Co-IP data validate binding of PAT1a to RME-6 and suggest the hinge region between the RasGAP and VPS9 domain as the interaction site (amino acids 561-1170). To determine the cellular localization of RME-6, Flag-tagged RME-6 was exogenously expressed in HeLa cells and the recombinant RME-6 was stained with an anti-Flag antibody (Fig. 5a). RME-6 showed a mostly cytoplasmic localization. However, co-expression of APP caused a clear redistribution of RME-6 to vesicular structures, to which APP and RME-6 colocalized (Fig. 5a) that were at least partially EEA1 positive (Fig. 5b). Similarly, also heterologously expressed HA-tagged PAT1a (PAT1a HA) and myc APP colocalized (Fig. 5c). Based on these data, we assumed that APP, PAT1a and RME-6 might form a tripartite complex. To address this point, we performed triple transfections of HeLa cells with expression constructs encoding myc APP, PAT1a HA and Flag RME-6. Interestingly, we observed a punctate colocalization of all three proteins (Fig. 5d). Altogether, our studies strongly suggest the Rab5 activator RME-6, as a novel PAT1a interacting protein, possibly forms a trimeric complex with APP.

### RME-6 knockdown influences clathrin-dependent APP endocytosis

**Fig. 6:**
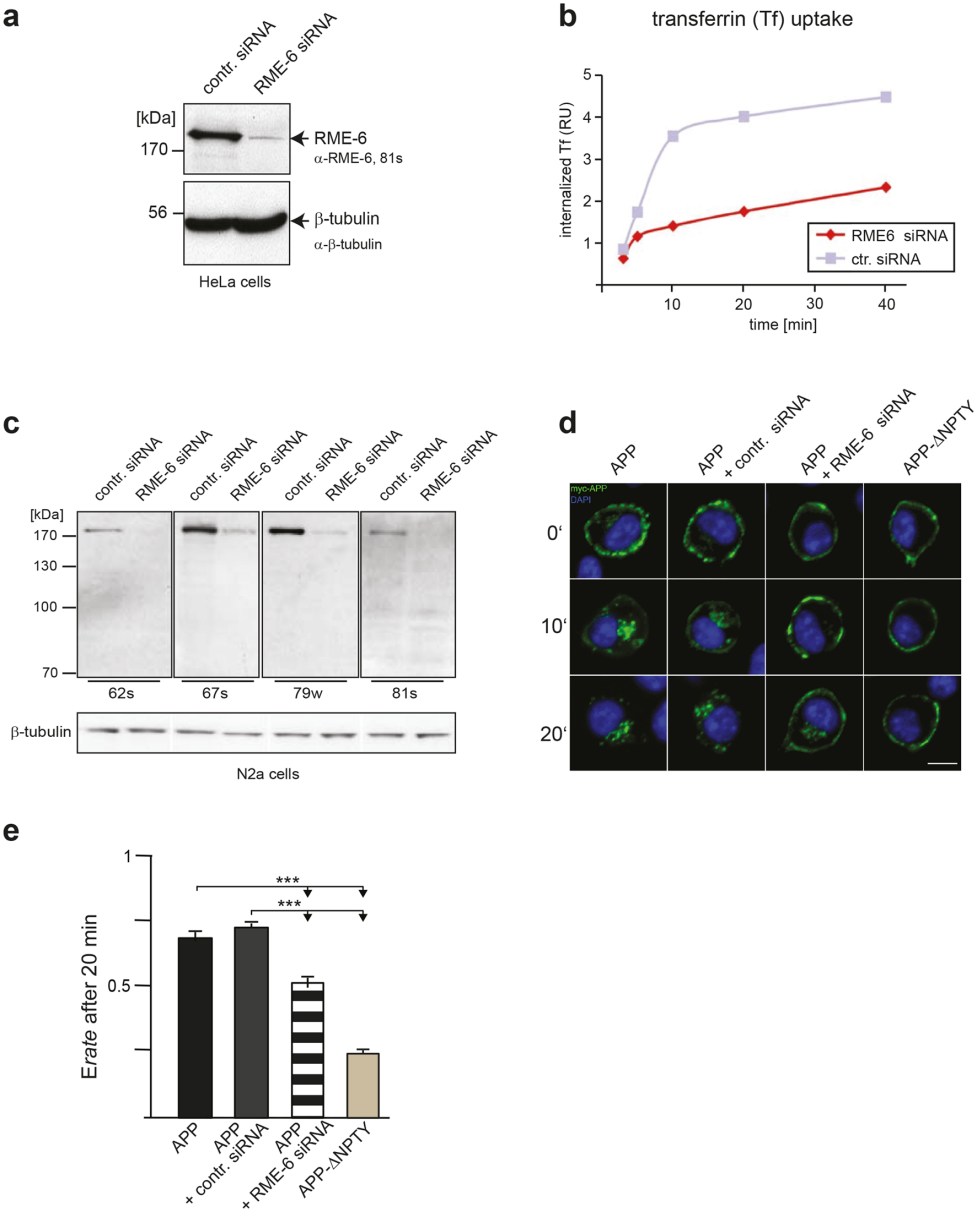
RME-6 affects APP endocytosis depending on the YTSI motif. **a** HeLa cells treated with RME-6 siRNA or control siRNA for 72 h, were subjected to Western blot analysis, using anti-RME-6 (81 s) and for control anti-β-tubulin antibodies. **b** HeLa cells treated for 72 h with control siRNA (ctr. siRNA) and siRNA directed against RME-6 were incubated with ruthenium-labeled transferrin for the indicated time periods, at 37 °C, to allow transferrin receptor (TR) uptake. The amount of endocytosed ruthenium-labeled transferrin was measured using Meso Scale Defense’s (MSD) technology. The relative amount of endocytosed ruthenium-labeled transferrin (relative units—RU) is presented with regard to a function of time (means ± SEM; *n* = 3) **c** Mouse monoclonal antibodies (62 s, 67 s, 79w, and 81 s) directed against the RasGAP-like domain of RME-6 were generated (see “Materials and methods”) and tested via Western blot analysis, using extracts of N2a cells treated with RME-6 siRNA or control siRNA. β-Tubulin staining served as loading control. **d** Influence of RME-6 on APP internalization via an antibody uptake assay. N2a cells expressing myc-APP, treated with siRNA directed against RME-6 or control siRNA, were incubated with mouse anti-myc antibody at 4 °C. 0, 10 or 20 min after shifting the cells to 37 °C (to facilitate endocytosis), they were fixed and stained by DAPI (blue) and for APP with an anti-mouse Alexa Fluor 488-conjugated secondary antibody. N2a cells expressing myc-tagged APP ΔNPTY served as controls. **e** For quantification, internal/total intensity ratios (*R*_Endo_) were determined after endocytosis for 20 min. The ratio (*R*_Endo_) of total to inner APP immunofluorescence signals was taken as quantitative measure of endocytosis rate. Bars represent mean values ± SEM; *t *test; comparing APP WT to APP ΔNPTY and control siRNA to RME-6 siRNA treated cells (*N* = 3, *n* > 30; ***p < 0.001)

To investigate whether RME-6 affects APP endocytosis, we decided to analyze the same in RME-6-depleted cells. For this purpose, we first generated different monoclonal antibodies against RME-6. Specificity of the antibodies from four different hybridoma cell clones (62 s, 67 s, 79w and 81 s) was tested. The immunoglobulin subclasses were identified to be IgG 2b + 1 for 62s; IgG 2b for 67s; IgG 1 for 79w and IgG 2b for 81s. Specificity and sensitivity of the Protein-G affinity-purified antibodies was investigated using N2a cells treated with RME-6-specific siRNA or non-functional siRNA (control siRNA) as control. 48 h after siRNA treatment, the cells were lysed and subjected to polyacrylamide gel electrophoresis (PAGE) and Western blot analysis. All four anti-RME-6 antibodies (67 s, 79w, 62 s and 81 s) recognized a single protein species at approximately 170 kDa (Fig. 6c). The signal was clearly reduced in lysates from cells treated with RME-6 siRNA, indicating specificity of the antibody. Minor low molecular weight bands were recognized by the different antibodies (Fig. 6c), which were likely due to low unspecific binding of the different antibodies, as the signals were not affected by treatment with the RME-6 siRNA (Fig. 6c). For loading control, anti-β-tubulin was used. Quantification of Western blot analyses revealed an RME-6 knockdown efficiency of approximately 90%. Further, testing of the antibodies in immunocytochemistry (IC) analyses revealed no specific signals (data not shown), indicating that the epitopes were masked under the tested IC conditions. The RME-6 siRNA was designed to target both, mouse and human RME-6. To validate this knockdown in human cells, we treated HeLa cells with the siRNA and in addition observed reduction of RME-6 levels by about 90% (Fig. 6a). Others reported that RME-6 functions as an activator of Rab5 [32, 46]. To explore the functional role of the mammalian RME-6 in Rab5-mediated endocytic transport, the internalization rate of biotinylated and ruthenium-labeled transferrin was measured in HeLa cells, transfected with siRNA specific for RME-6 or control siRNA. 74 h post-transfection, cells were incubated with the labeled transferrin at 37 °C for different time periods (5, 10, 20 and 40 min), washed and lysed. The amount of internalized ruthenium-labeled transferrin was measured in triplicate using a SECTOR Imager 6000 (Meso Scale Discovery). The amount of internalized transferrin was standardized with respect to the total protein concentration of the lysate and expressed as relative units (Fig. 6b). Strikingly, the knockdown of RME-6 markedly inhibited transferrin internalization.

To investigate whether RME-6 is also involved in APP endocytosis, an immunofluorescence-based APP antibody uptake assay was performed. N2a cells expressing N-terminally myc-tagged APP were treated with RME-6-specific siRNA or non-functional siRNA (control siRNA) for 48 h (Fig. 6d). In addition, we used cells expressing APP ΔNPTY as a positive control. Quantification of the signal intensity of endocytosed to total APP ratio revealed a significant reduction of the APP endocytosis rate upon RME-6 knockdown, when compared to non-treated or control siRNA-treated cells (Fig. 6e). Notably, the inhibition of APP endocytosis was less pronounced than the inhibition observed for APP lacking the NPTY motif. Together, these results clearly indicate that RME-6 is involved in APP endocytosis.

### Functional interaction of RME-6 and PAT1a in APP endocytosis depends on the YTSI motif

**Fig. 7:**
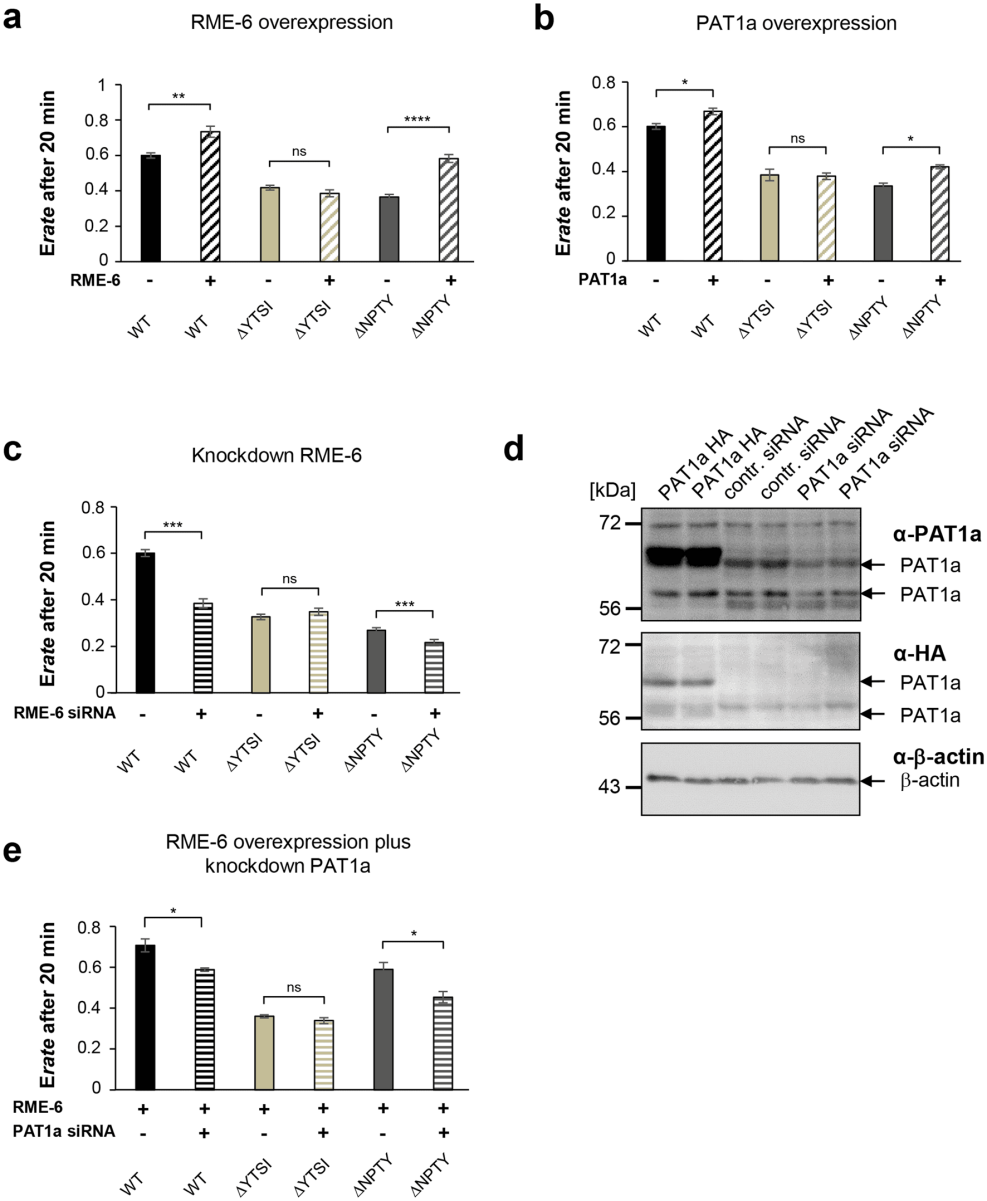
Functional interaction of RME-6 and PAT1a in APP endocytosis, depending on the YTSI motif. (a, b) N2a cells co-expressing myc APP or different myc-tagged APP mutants (ΔYTSI and ΔNPTY) along with **a** RME-6 or **b** PAT1a were subjected to an antibody uptake assay. Quantification of internal/total intensity ratios after 20 min is shown. **c** Alternatively, cells expressing myc-APP, APP ΔYTSI or APP ΔNPTY were treated with siRNA directed against RME-6, 72 h prior to initiation of the antibody uptake assay. Quantification of internal/total intensity ratios after 20 min is shown. Bars represent mean values ± SEM; ANOVA; comparing treated to non-treated cells (*N* ≥ 2, *n* ≥ 12; ***p < 0.001). **d** N2a cells were transfected with HA-tagged PAT1a cDNA or treated with siRNA directed against PAT1a, or for control with scrambled siRNA, for 72 h. Lysates were subjected to Western blot analysis using anti-PAT1a, anti-HA, as well as anti-β-actin antibodies. **e** N2a cells co-expressing myc APP or different myc-tagged APP mutants (ΔYTSI and ΔNPTY), along with RME-6, were treated with PAT1a siRNA (+) or control siRNA (−), and were subjected to an antibody uptake assay. Quantification of internal/total intensity ratios after 20 min is shown. Bars represent mean values ± SEM; ANOVA, Tukey’s HSD post hoc test, comparing treated to non-treated cells (*N* ≥ 2, *n* ≥ 12; ***p < 0.001)

Based on our data, we assumed that the increased levels of RME-6 and PAT1a would promote APP endocytosis. To test this assumption, we subjected N2a cells expressing APP together with either RME-6 or PAT1a, to an antibody uptake assay. As expected, overexpression of RME-6 as well as PAT1a caused significantly increased APP endocytosis (Fig. 7a, b). Interestingly, the RME-6 and PAT1a-mediated APP endocytosis rate was only increased in cells expressing APP WT and APP ΔNPTY, but not in those expressing APP ΔYTSI. Notably, the effect of RME-6 overexpression had a larger impact on the endocytosis of APP ΔNPTY in comparison to APP WT. This corroborates our assumption that the promoting influence of RME-6 and PAT1a depends on the YTSI motif. Moreover, we tested if the previously observed inhibition of APP endocytosis upon knocking down RME-6 (Fig. 6d, e) might depend on the YTSI motif. To address this point, we investigated if a knockdown of RME-6 aggravates the endocytosis rate of APP, APP ΔNTPY and APP ΔYTSI. Interestingly, the endocytosis rate of APP ΔNTPY was further decreased by about 15% when the cells were treated with RME-6 siRNA. In contrast, the internalization rate observed for APP ΔYTSI was unchanged after depleting RME-6 (Fig. 7c). Finally, we intended to test for a functional interaction between RME-6 and PAT1a, by simultaneously knocking down PAT1a while overexpressing RME-6. We first validated the knockdown efficiency of PAT1a siRNA [38] in N2a cells by performing Western blot analysis (Fig. 7d). Then, we performed an antibody uptake assay with APP WT, as well as APP ΔNTPY and APP ΔYTSI, under RME-6 overexpression and PAT1a knockdown conditions. Despite elevated RME-6 levels resulting in increased APP endocytosis (Fig. 7a), simultaneous knockdown of PAT1a significantly reversed this effect. Under identical conditions, APP ΔNTPY behaved similar to APP WT. In contrast, no change in endocytosis rate was observed in APP ΔYTSI (Fig. 7e).

Together, these data clearly suggest a functional interaction of RME-6 and PAT1a in APP endocytosis, depending on the YTSI motif.

## Discussion

Here, we show that APP is taken in from the plasma membrane, involving the cytosolic domain with two endocytosis signals, the YTSI [8, 9] and the NPTY [8, 19] motif and show the contribution of the Rab5 activator RME-6 and PAT1a in APP endocytosis.

We observed by direct comparison of APP deletion mutants lacking the YTSI (APP ΔYTSI) or the NPTY motif (APP ΔNPTY) that deletion of each motif significantly decreased APP endosomal localization (Fig. 1), as well as endocytosis rates to about 40 and 30%, respectively (Fig. 2). This indicates that besides the well-established involvement of the NPTY sequence, the YTSI motif is also required for efficient APP endocytosis. Moreover, we observed increased APP endocytosis in the presence of elevated PAT1a levels (Fig. 7). These results are in line with a study showing that downregulation of PAT1a, which binds the YTSI motif, leads to increased APP levels at the cell surface [54] and a more recent analysis, showing colocalization of EEA1 and PAT1a [38]. The YTSI motif of APP conforms to the tyrosine-dependent YXXФ sequence and might therefore be able to induce CME by direct binding to the μ2 subunit of AP2 [55, 56]. However, it has been reported that the μ2 subunit of AP2 does not interact with the YTSI motif of APP [12, 57]. Remarkably, purely endocytic YXXΦ signals are most often located 10–40 residues from the transmembrane domain, whereas lysosomal targeting YXXΦ signals are conspicuous by their presence at 6–9 residues from the transmembrane domain [21]. Here, the binding site also covers most of the N-terminal amino acids directly at the end of the transmembrane domain (Fig. 1). These data indicate that the YTSI motif in APP represents no typical YXXΦ signal.

We found that the YTSI-binding protein PAT1a interacts with the Rab5 GDP/GTP exchange factor RME-6, as validated by the two-hybrid system (Fig. 3), GST pulldown analyses (Fig. 4), co-immunoprecipitation experiments (Fig. 4) and colocalization studies (Fig. 5). These data suggest that APP, PAT1a and RME-6 form a tripartite complex.

RME-6 has two conserved domains, an N-terminal RasGAP-like domain and a C-terminal VPS9 domain. RME-6 was reported to activate Rab5 via its VPS9 domain [58] and to alter receptor-mediated endocytosis by regulating clathrin-coated vesicle-mediated transport, from the clathrin-coated pit (CCP) [59, 60] to early endosomes, as well as homotypic early endosome fusion (for review see [61]). Besides this function, Rab5 also affects protein sorting within endosomes [62] and regulates the motility of early endosomes along microtubules [63]. Consistent with the function of RME-6 in clathrin-dependent endocytosis, we found that reduced RME-6 protein levels led to a significant reduction of ruthenium-labeled transferrin internalization in HeLa cells (Fig. 6), suggesting an essential function of RME-6 for normal transferrin receptor (TR) endocytosis. In line with this, Sato et al. reported similar effects in *C. elegans* rme-6 mutants, exhibiting high-level accumulation of a fluid-phase endocytosis marker and a marker for clathrin-dependent endocytosis in the body cavity of the worm [46]. Co-staining of recombinant RME-6 with the early endosomal marker EEA1 showed a segregation of RME-6 and EEA1-positive structures (Fig. 5a), which is in agreement with previous studies in *C. elegans*, showing that RME-6 does not colocalize with early endosomal marker [46]. In line with this, a recent study by Semerdjieva and co-workers reported that RME-6 binds the α-ear of AP2 and replaces AP2-associated kinase 1 (AAK1), which leads to μ2 dephosphorylation and thus weaker cargo–μ2 interaction. Activated Rab5 recruits downstream effector like protein phosphatases, which mediate PI(4,5)P2 turnover, and thereby facilitate AP2 uncoating [64]. Notably, GEF activity of full-length RME-6 requires binding to the α-ear of AP2, which ensures its functionality only in clathrin-coated vesicles (CCVs) [64]. As we found that PAT1a binds stronger to deletion constructs of RME-6, lacking either the VPS9 or GAP domain, it appears reasonable that PAT1a might only interact with RME-6, when it is bound in addition to other factors, possibly uncovering the PAT1a interaction domain in the hinge region (Fig. 4). Based on the reported function of RME-6 in CCV uncoating, it is tempting to speculate that RME-6, implicating the YTSI motif and PAT1a, might have a similar function in APP endocytosis. Consistent with this assumption, we found that co-expression of APP and RME-6 causes a redistribution of RME-6 to APP containing vesicles, colocalizing at least partially with EEA1 (Fig. 5b). Furthermore, overexpression or knockdown of RME-6 increases/decreases APP endocytosis. Moreover, the endocytosis rate of APP ΔNPTY was influenced similarly, whereas APP ΔYTSI was not affected under the same conditions (Fig. 6, 7).

Our data show that depleting RME-6 markedly reduces transferrin endocytosis, the canonical marker of clathrin-mediated endocytosis [65], whereas the reduction of APP endocytosis was less pronounced. Taking into account that CME is regulated in different steps (initiation, cargo selection, maturation, and fission) by the major coat proteins and a myriad of endocytic accessory proteins and phosphatidylinositol lipids [66], it appears reasonable that APP might go through clathrin-mediated endocytosis by using a set of regulators other than the transferrin receptor. However, as PAT1a was reported to also affect transferrin receptor recycling in a Rab5-dependent manner [38], later steps in the recycling pathway of the transferrin receptor might also contribute to the impact of RME-6 depletion. Alternatively, the difference might be explained by a distinct function of RME-6 in APP clathrin-independent mechanisms.

**Fig. 8:**
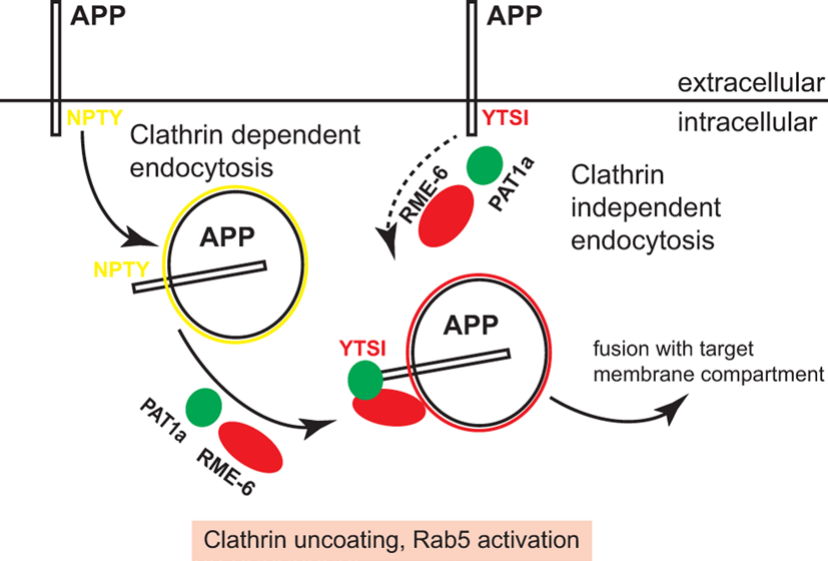
Model of RME-6/PAT1a function in APP endocytosis. APP is mainly endocytosed by clathrin-dependent (CME) and to a minor extent clathrin-independent endocytosis. CME depends on the NPTY motif, linking the AP2 complex that mediates clathrin coat formation and budding of the vesicle [74]. After scission the clathrin-coated vesicle gets uncoated, a process possibly involving RME-6, as reported by others [31, 63]. Alternatively, APP clathrin-independent endocytosis depends on RME-6, recruited by PAT1a interacting with the YTSI motif, as supported by our data, showing an additive function of the NPTY and YTSI motif in APP endocytosis

We observed that the internalization deficit of APP ΔNPTY was clearly increased by additional deletion of the YTSI motif or a knockdown of RME-6 (Figs. 1, 2, 7). This argues for involvement of the NPTY motif and RME-6, as well as the YTSI motif in different APP endocytosis pathways (Fig. 8). This assumption is further corroborated by the finding that PAT1a as well as RME-6 overexpression only increased APP WT and APP ΔNPTY endocytosis, but not APP ΔYTSI internalization. In addition, we provide evidence for a functional interaction between RME-6 and PAT1a depending on the YTSI motif (Fig. 7). Thus, the NPTY motif might be involved in clathrin-dependent endocytosis, whereas RME-6 and PAT1a more likely contribute via the YTSI motif to alternative pathways, such as caveolin-mediated endocytosis of APP. Notably, caveolin-dependent endocytosis has been reported for APP before [7] and this internalization was documented to involve activation of Rab5 [67]. However, alternative explanations are also possible. The deletion of the NPTY motif might not block clathrin-dependent endocytosis completely, as AP-2 function might be bypassed by alternative adaptors, as postulated for low density lipoprotein (LDL) receptors under certain conditions [68]. Thus, RME-6 and the YTSI motif either function in clathrin-dependent or alternative AP-2-independent APP endocytosis pathways.

Despite the fact that endocytosis is a very fast process requiring only a few minutes [69], we detected in our antibody uptake assay, after a few minutes, very low levels of intracellular APP immunoreactivity that continuously increased over a time period of 20 min. This discrepancy is likely due to two major limitations in our experimental conditions. Firstly, the spatial resolution of our microscopic analysis allows only differentiation of particles with distances > 200 nm. Thus, we likely do not detect just endocytosed vesicles, but instead only distinguish vesicles clearly separated from the plasma membrane. Secondly, we likely detect intracellular immunoreactivity only above a certain threshold, corresponding to multiple APP molecules. Calculations of retrograde transported vesicles revealed 100–150 APP molecules per transport vesicle [70]. Future studies with higher sensitivity and higher spatial resolutions would be highly interesting, as this would allow to decipher the endocytosis steps mediated by the NPTY and YTSI motif-associated proteins in more detail.

In line with other studies, we show that apart from the two major sorting signals (NPTY, YTSI), APP endocytosis is also regulated by ubiquitination, as studied in detail for the EGF receptor [71–73]. However, at least in N2a cells, used in our study, the endocytosis rate of the ubiquitination-deficient mutant, APP 5R was affected only moderately (Fig. 2). Possibly, later steps of APP sorting to distinct targets, including autophagosomes, lysosomes or late endosomes, are more dominantly controlled by ubiquitination [50, 74].

Altogether, our findings give novel insights into the molecular mechanisms underlying APP endocytosis by highlighting a function of PAT1a as a scaffolding protein and interacting with the APP YTSI motif, promoting APP endocytosis, presumably by RME-6-dependent activation of Rab5.

## Electronic supplementary material

Below is the link to the electronic supplementary material.

Supplementary file1 (PDF 1038 kb)

Supplementary file2 (PNG 107 kb)

Supplementary file3 (PDF 39 kb)

Supplementary file4 (AVI 28130 kb)

Supplementary file5 (AVI 32507 kb)

Supplementary file6 (AVI 32810 kb)

Supplementary file7 (AVI 32709 kb)

Supplementary file8 (AVI 32557 kb)

Supplementary file9 (AVI 32153 kb)

Supplementary file10 (AVI 32608 kb)
