# Supplementary material for: The Rab5 activator RME-6 is required for amyloid precursor protein endocytosis depending on the YTSI motif
Source: Cell Mol Life Sci. 2020 Feb 17;77(24):5223–42. doi: 10.1007/s00018-020-03467-1 (PMC7671991; doi:10.1007/s00018-020-03467-1)
Supplement: Supplementary file 1 — Supplementary file1 (PDF 1038 kb) [file 18_2020_3467_MOESM1_ESM.pdf]

ESM 1

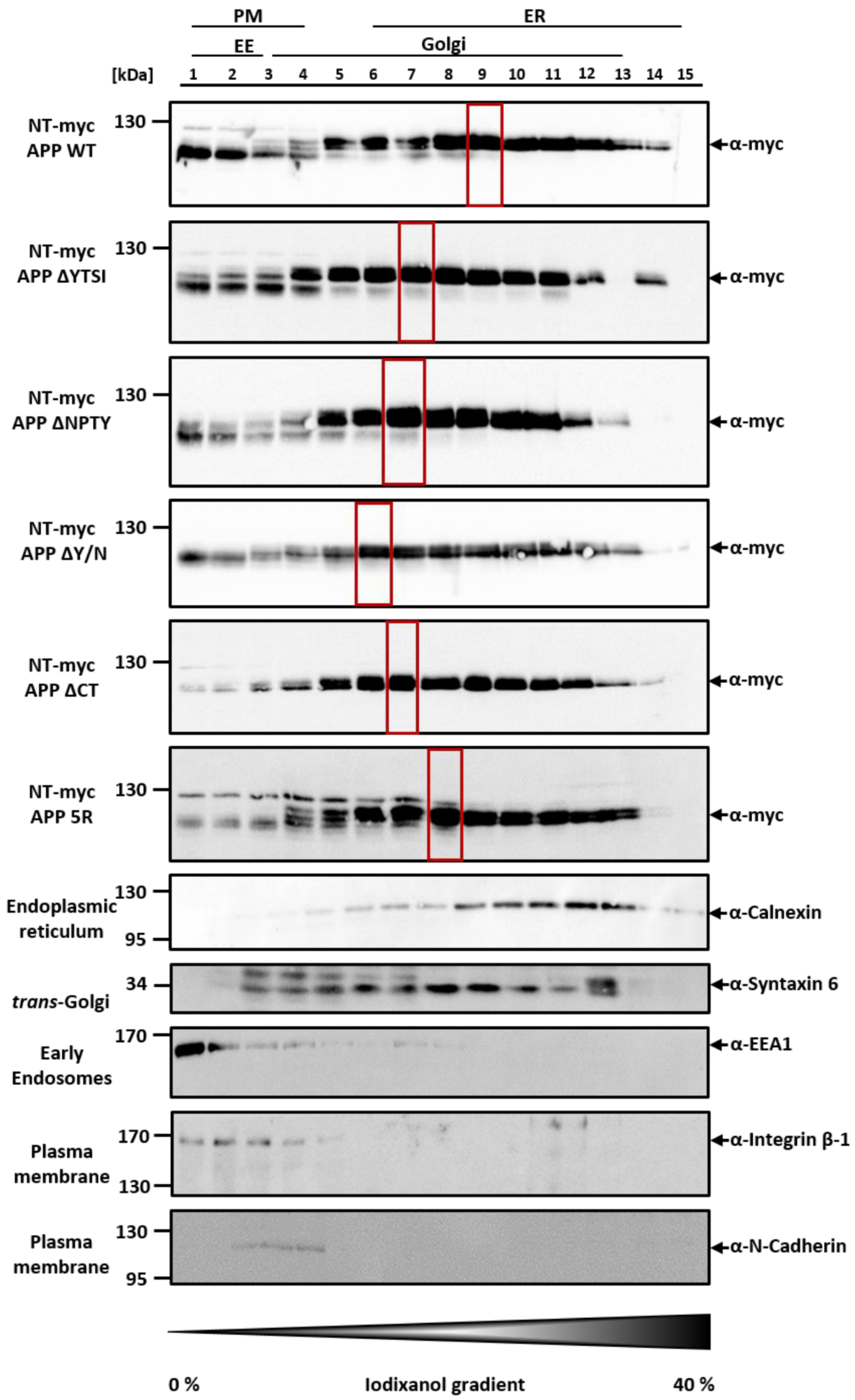

ESM 1a: Subcellular fractionation of APP WT and APP endocytosis deficient mutants. N2a cells were transiently transfected with myc APP WT and the mutants ΔYTSI, ΔNPTY, ΔY/N, ΔCT or 5R. 18 h later, the cell homogenates were fractionated via a continuous 0–40% iodixanol (Optiprep) gradient. Equal volumes of the samples were analyzed. Western blot analysis using α-myc antibody (9E10) shows the distribution of heterologously expressed APP and indicated APP mutants. Anti-Calnexin antibody was used to detect the ER, α-Syntaxin 6 for the *trans*-Golgi apparatus, α-EEA1 to reveal early endosomes, Integrin-β-1 and N-Cadherin as a marker for the plasma membrane. The red rectangulars indicate the quantified peak signal for each APP construct analyzed. Note, that there is a shift to the left compared to APP WT for all APP endocytosis deficient mutants.

ESM 1

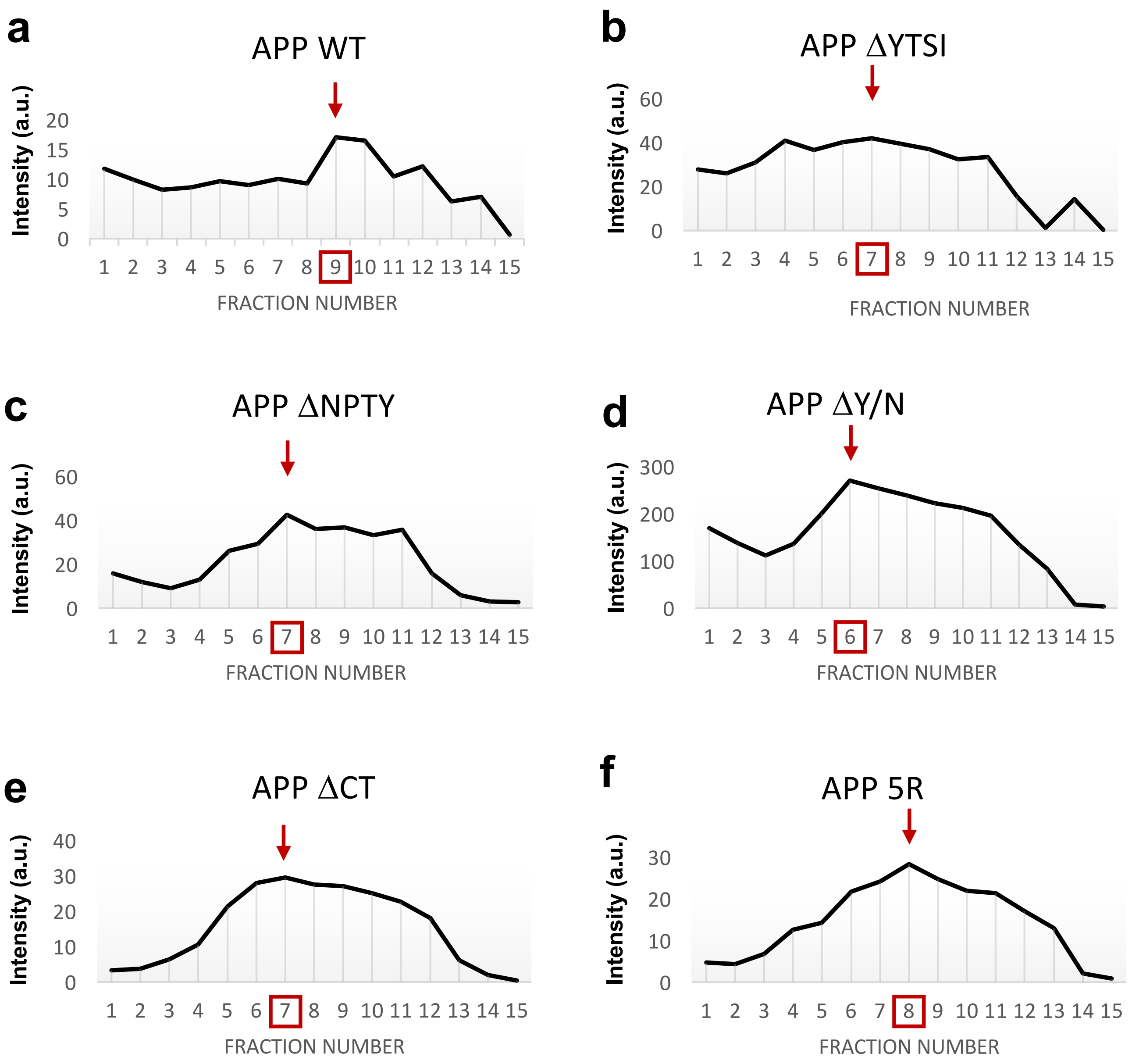

ESM 1b: Quantification of the subcellular fractionation of APP WT and APP endocytosis deficient mutants. Western blots shown were evaluated via densitometric analysis (Image J). The obtained data revealed that there is a peak shift to the left compared to APP WT, for all APP endocytosis deficient mutants.
