# Supplementary figures and images for: The Rab5 activator RME-6 is required for amyloid precursor protein endocytosis depending on the YTSI motif
Source: Cell Mol Life Sci. 2020 Feb 17;77(24):5223–42. doi: 10.1007/s00018-020-03467-1 (PMC7671991; doi:10.1007/s00018-020-03467-1)

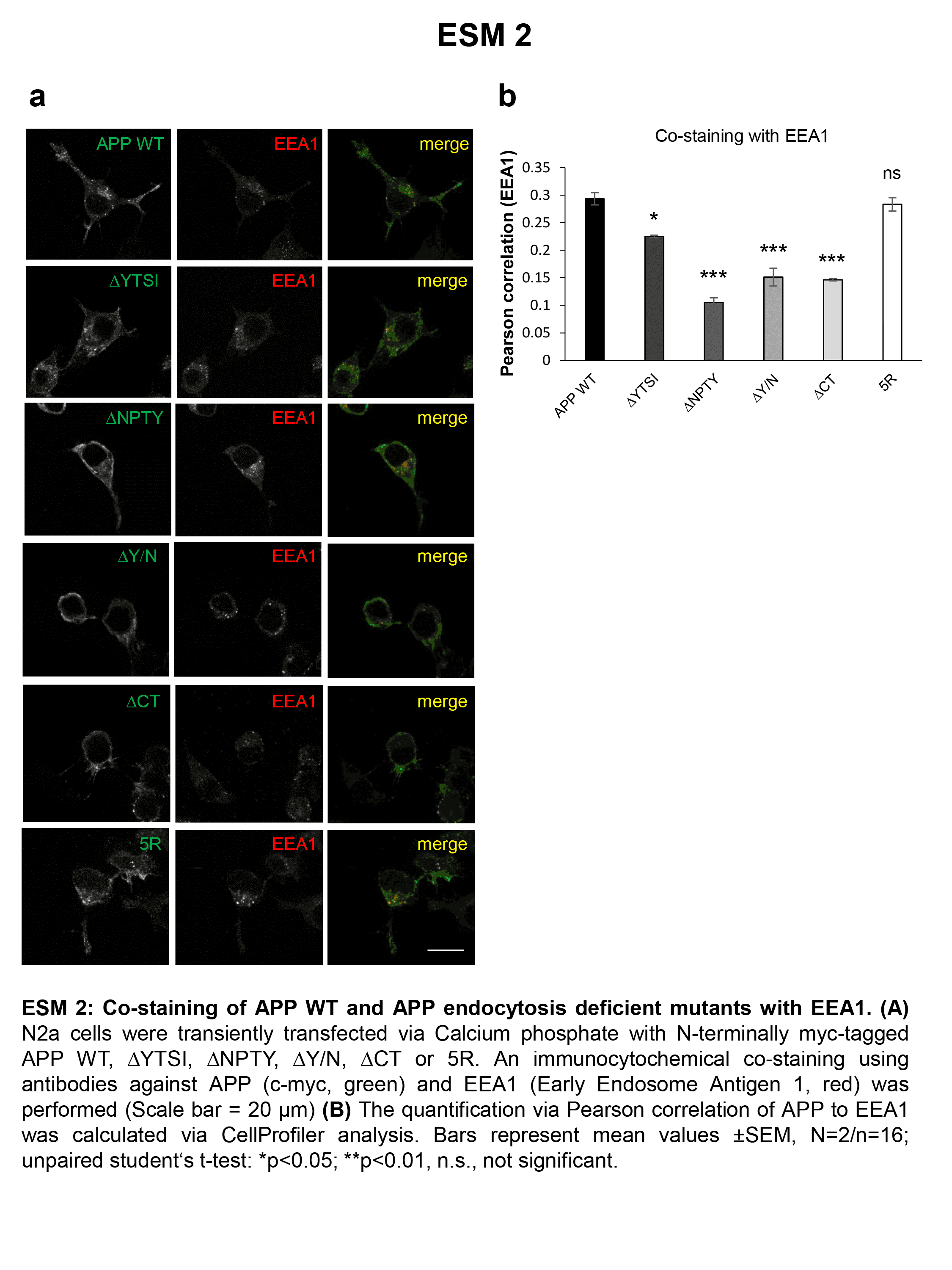

Supplement: Supplementary file 2 — Supplementary file2 (PNG 107 kb) [file 18_2020_3467_MOESM2_ESM.png]
