# Supplementary material for: The Rab5 activator RME-6 is required for amyloid precursor protein endocytosis depending on the YTSI motif
Source: Cell Mol Life Sci. 2020 Feb 17;77(24):5223–42. doi: 10.1007/s00018-020-03467-1 (PMC7671991; doi:10.1007/s00018-020-03467-1)
Supplement: Supplementary file 3 — Supplementary file3 (PDF 39 kb) [file 18_2020_3467_MOESM3_ESM.pdf]

ESM 3

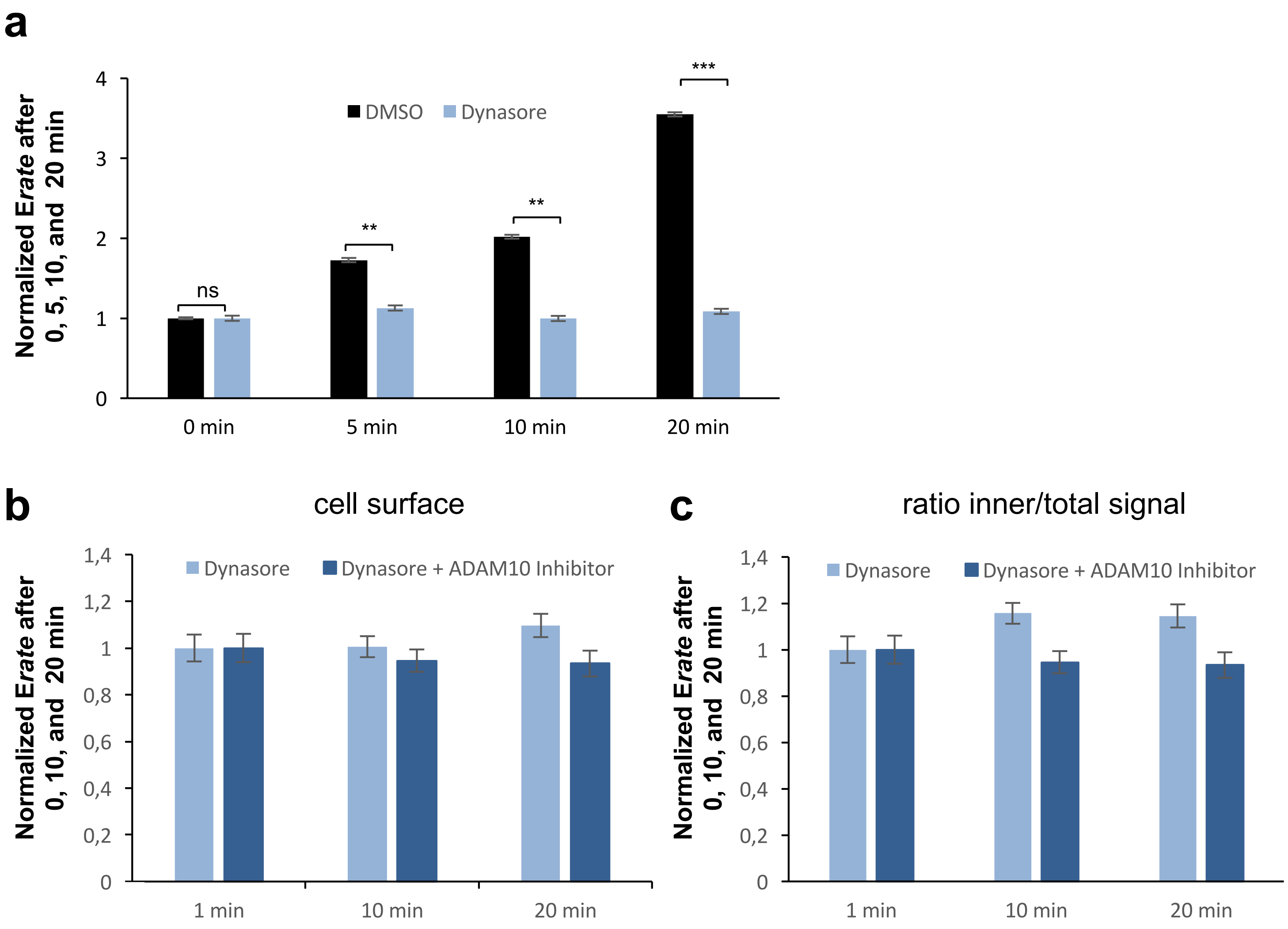

ESM 3: Dynasore treatment of APP WT. N2a cells were transiently transfected with myc APP WT. **(a)** The cells were loaded with 80 µM of the inhibitor of endocytosis, Dynasore or the vehicle control DMSO in DMEM media without FBS for 30 min at 37 °C. An antibody uptake assay was performed followed by antibody internalization at four different time points (0, 5, 10, and 20 min). Immunolabelling was carried out with α-c-myc antibody for cell surface APP at 4 °C, which included DMSO or Dynasore treatment (80 µM). Then, cells were allowed to endocytose antibody-labeled APP for 5, 10, and 20 min at 37 °C using prewarmed N2a cell culture media including either DMSO or 80 µM Dynasore. After fixation, non-permeabilized cells were stained with a secondary antibody (Alexa Fluor-488) to visualize surface APP. Afterwards, cells were permeabilized and internalized APP was labeled with a secondary antibody (Alexa Fluor-594). Internal/total intensity ratio of densitometric measurements was analyzed at the different endocytosis time points. Bars *are* representing mean values ± SEM; (N=2/n=12); one-way ANOVA with Tukey's HSD post hoc test *$p < 0.05$, **$p < 0.01$, ***$p < 0.001$. **(b, (c)** The cells were loaded with 80 µM Dynasore or Dynasore plus ADAM10 inhibitor GI254023X (10 µM) in DMEM media without FBS for 30 min at 37 °C. The treatment was continued during the myc-488 antibody labeling step at 4 °C and the 20 min Live cell recording at 37 °C (see above). Live cell recordings of 1, 10 and 20 minutes were analyzed with ImageJ. Signals after 1 min were normalized to 1 and the SEM is given. **(b)** The signal of cell surface APP was determined over a time span of 20 min, showing no significant changes. **(c)** The ratio of intracellular versus cell surface APP of the same cells did not show any significant changes after Dynasore or Dynasore plus ADAM10 inhibitor treatment. (N=2/n=7); one-way ANOVA with Tukey's HSD post hoc test *$p < 0.05$.
